# Supplementary material for: Psychostimulants and opioids differentially influence the epigenetic modification of histone acetyltransferase and histone deacetylase in astrocytes
Source: PLoS One. 2021 Jun 11;16(6):e0252895. doi: 10.1371/journal.pone.0252895 (PMC8195369; doi:10.1371/journal.pone.0252895)

HDAC1 with marker

Human Primary Astrocyte Sample
Fig. 1 A
Image captured by C300 azure biosystems
BIORAD: Clarity Western ECL Substrate

Primary Antibody dilution 1:1000
Secondary Antibody dilution 1:5000

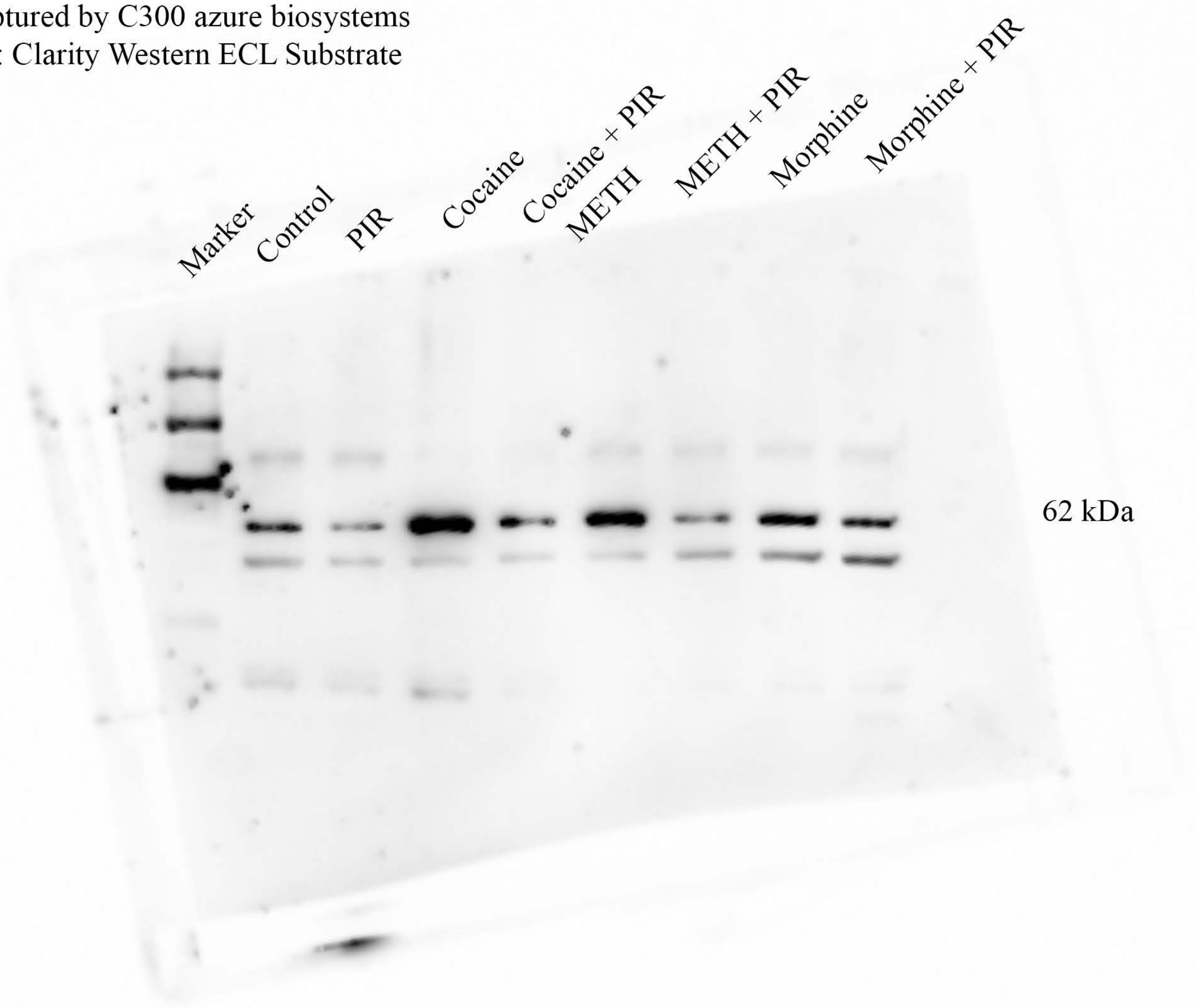

GAPDH for HDAC 1
Human Primary Astrocyte Sample
Fig. 1 A
Image captured by C300 azure biosystems
BIORAD: Clarity Western ECL Substrate

Primary Antibody dilution 1:1000
Secondary Antibody dilution 1:5000

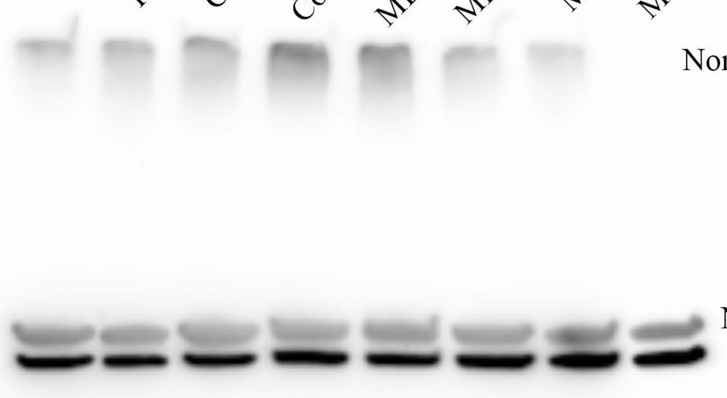

HDAC2 with marker
Human Primary Astrocyte Sample   Primary Antibody dilution 1:1000
Fig. 1 B   Secondary Antibody dilution 1:5000
Image captured by C300 azure biosystems
BIORAD: Clarity Western ECL Substrate

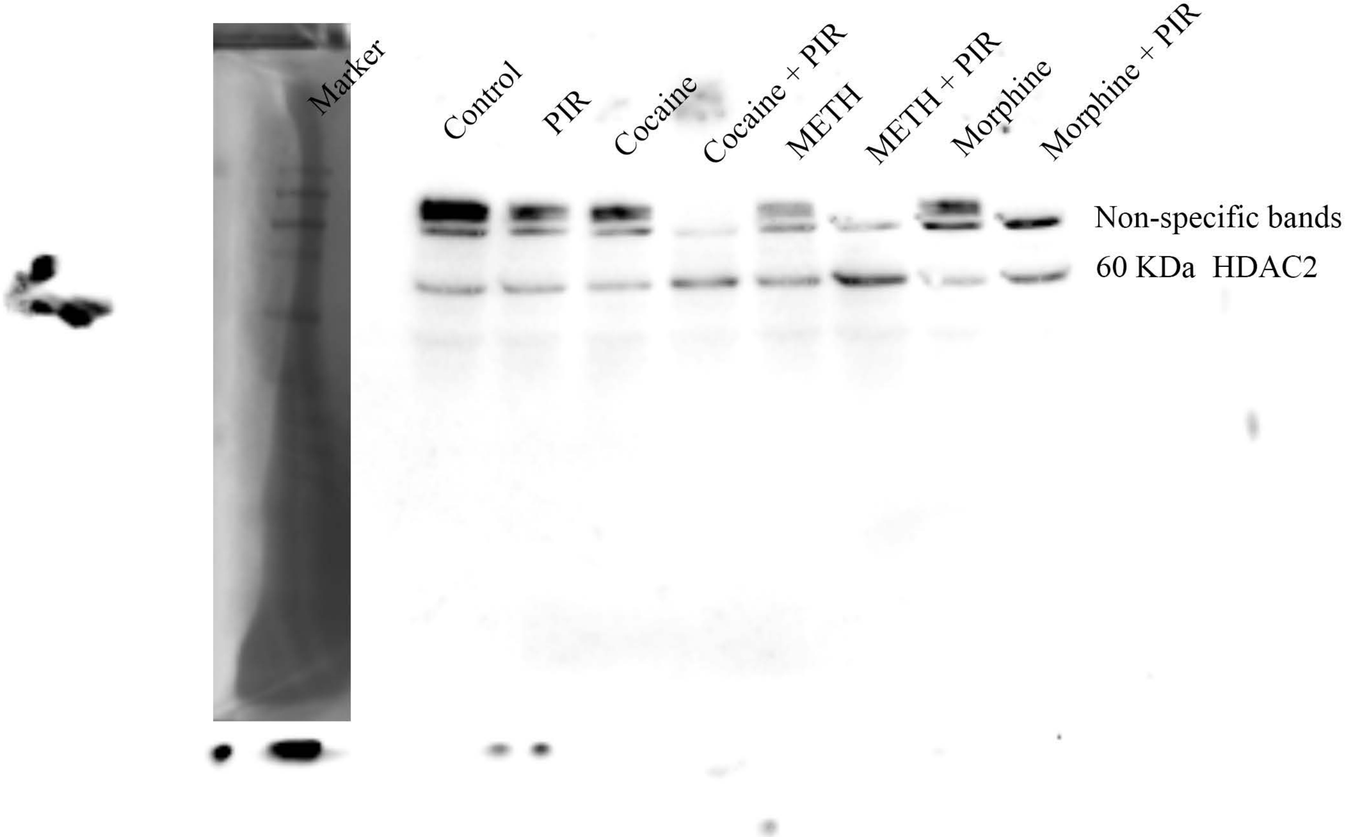

GAPDH for HDAC 2
Human Primary Astrocyte Sample		Primary Antibody dilution 1:1000
Fig. 1 B		Secondary Antibody dilution 1:5000
Image captured by C300 azure biosystems
BIORAD: Clarity Western ECL Substrate

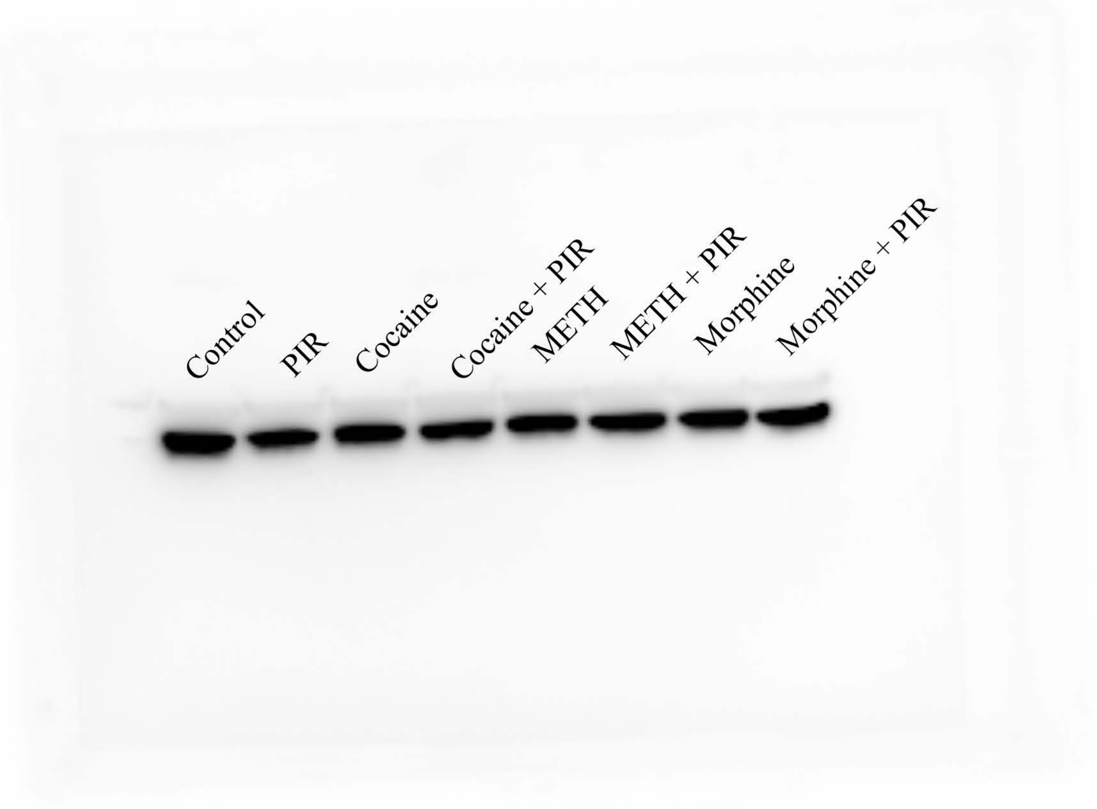

HDAC 3

Human Primary Astrocyte Sample — Primary Antibody dilution 1:1000

Fig. 1 C — Secondary Antibody dilution 1:5000

Image captured by C300 azure biosystems

BIORAD: Clarity Western ECL Substrate

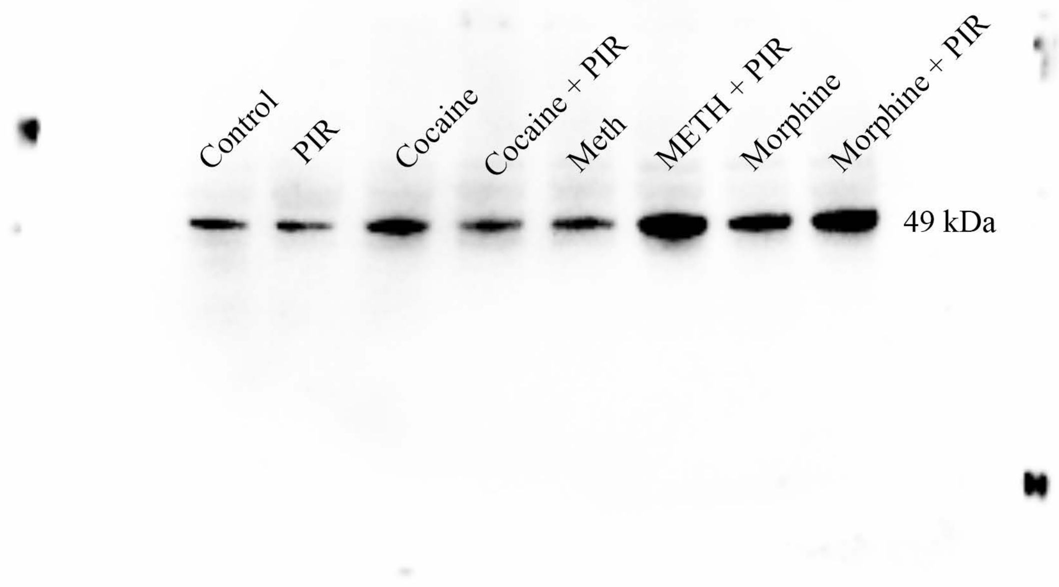

HDAC3 with marker

Human Primary Astrocyte Sample

Primary Antibody dilution 1:1000

Fig. 1 C

Secondary Antibody dilution 1:5000

Image captured by C300 azure biosystems

BIORAD: Clarity Western ECL Substrate

`

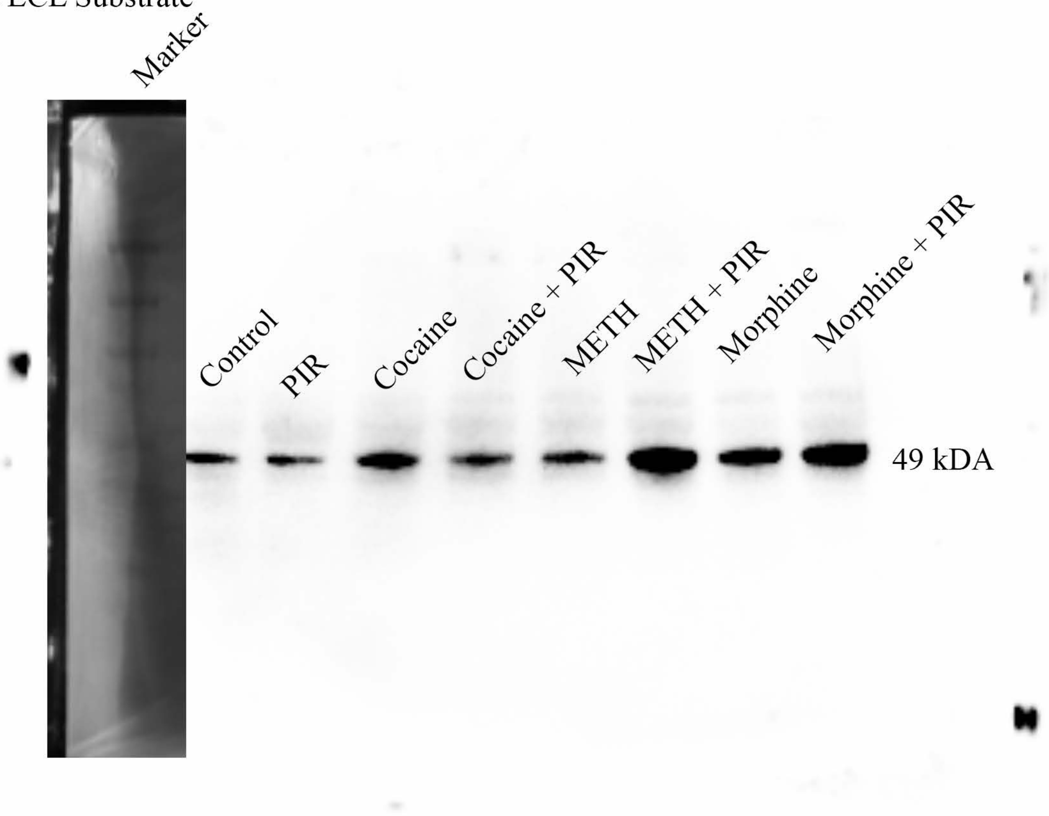

GAPDH for HDAC3
Human Primary Astrocyte Sample
Primary Antibody dilution 1:1000
Fig. 1 C
Secondary Antibody dilution 1:5000
Image captured by C300 azure biosystems
BIORAD: Clarity Western ECL Substrate

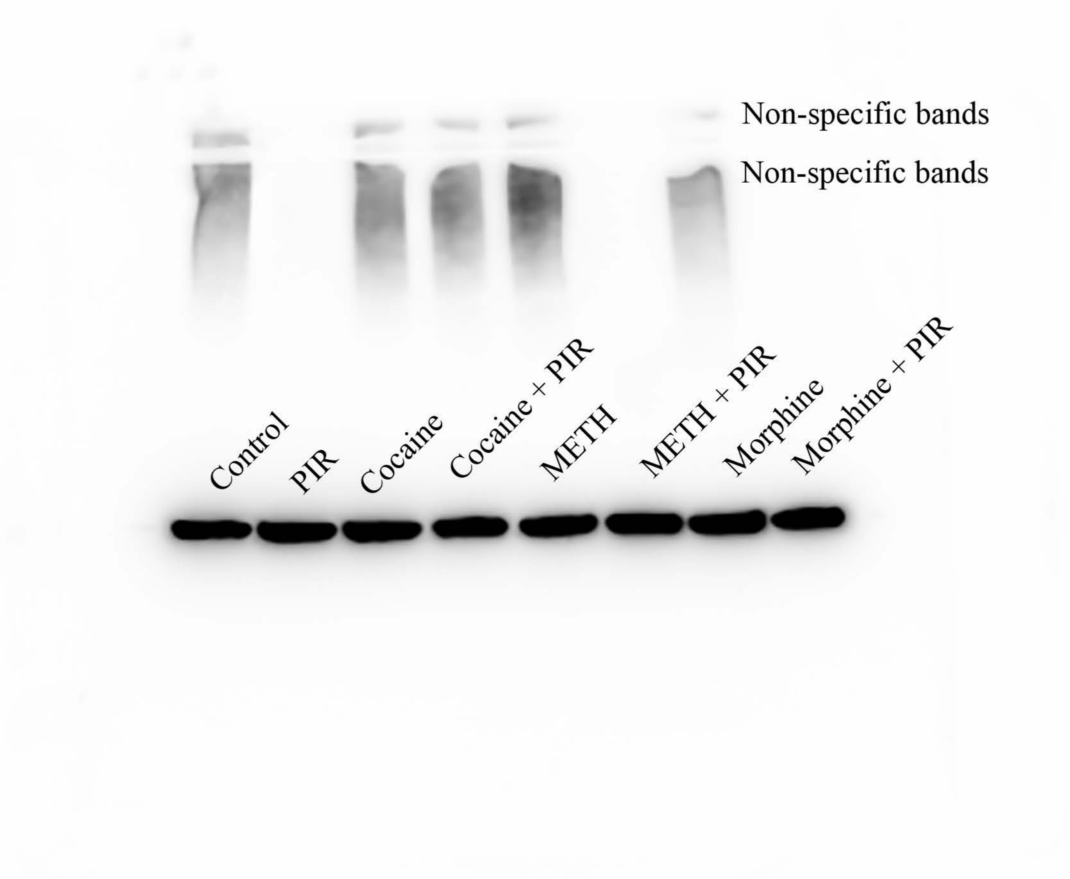

HDAC 4
Human Primary Astrocyte Sample	Primary Antibody dilution 1:1000
Fig. 2 A	Secondary Antibody dilution 1:5000
Image captured by C300 azure biosystems
BIORAD: Clarity Western ECL Substrate

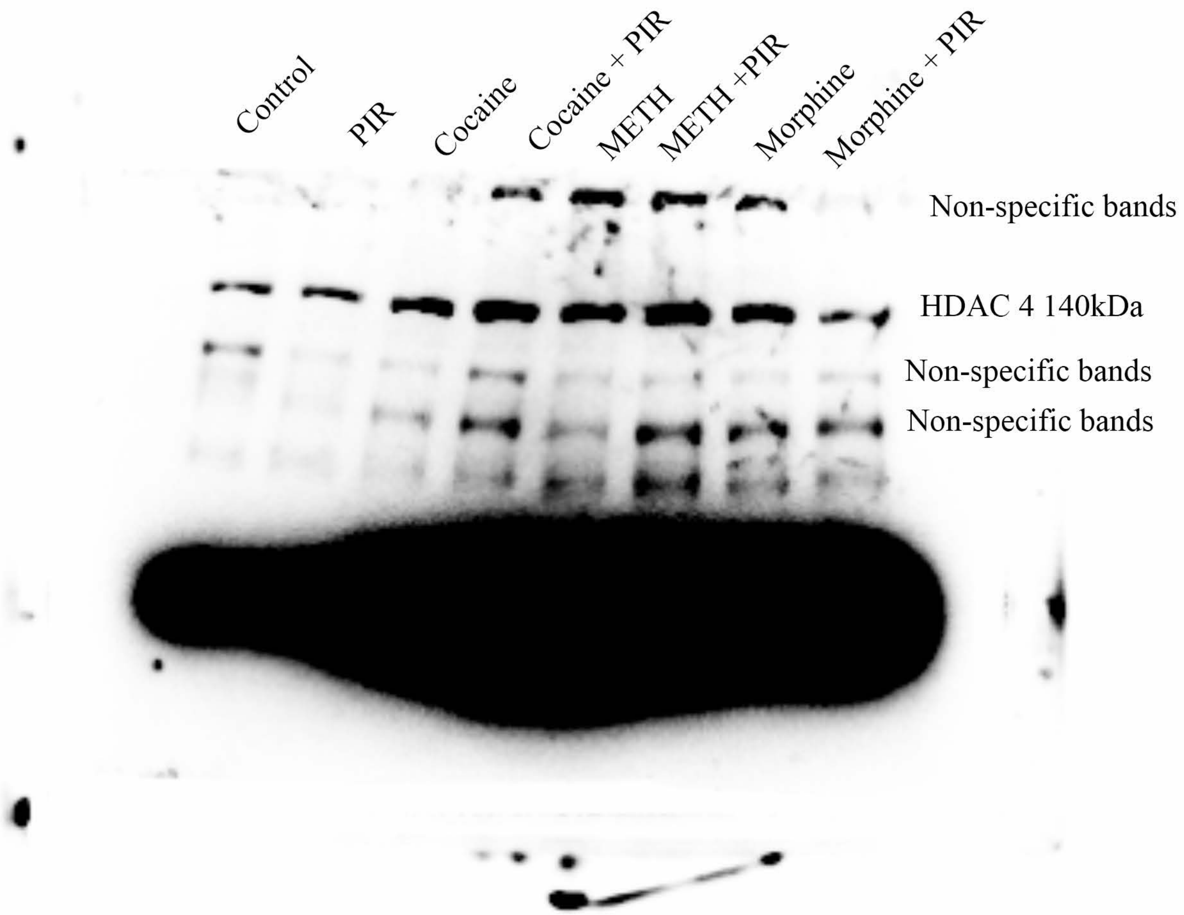

HDAC 4 with marker
Human Primary Astrocyte Sample
Fig. 2 A
Image captured by C300 azure biosystems
BIORAD: Clarity Western ECL Substrate

Primary Antibody dilution 1:1000
Secondary Antibody dilution 1:5000

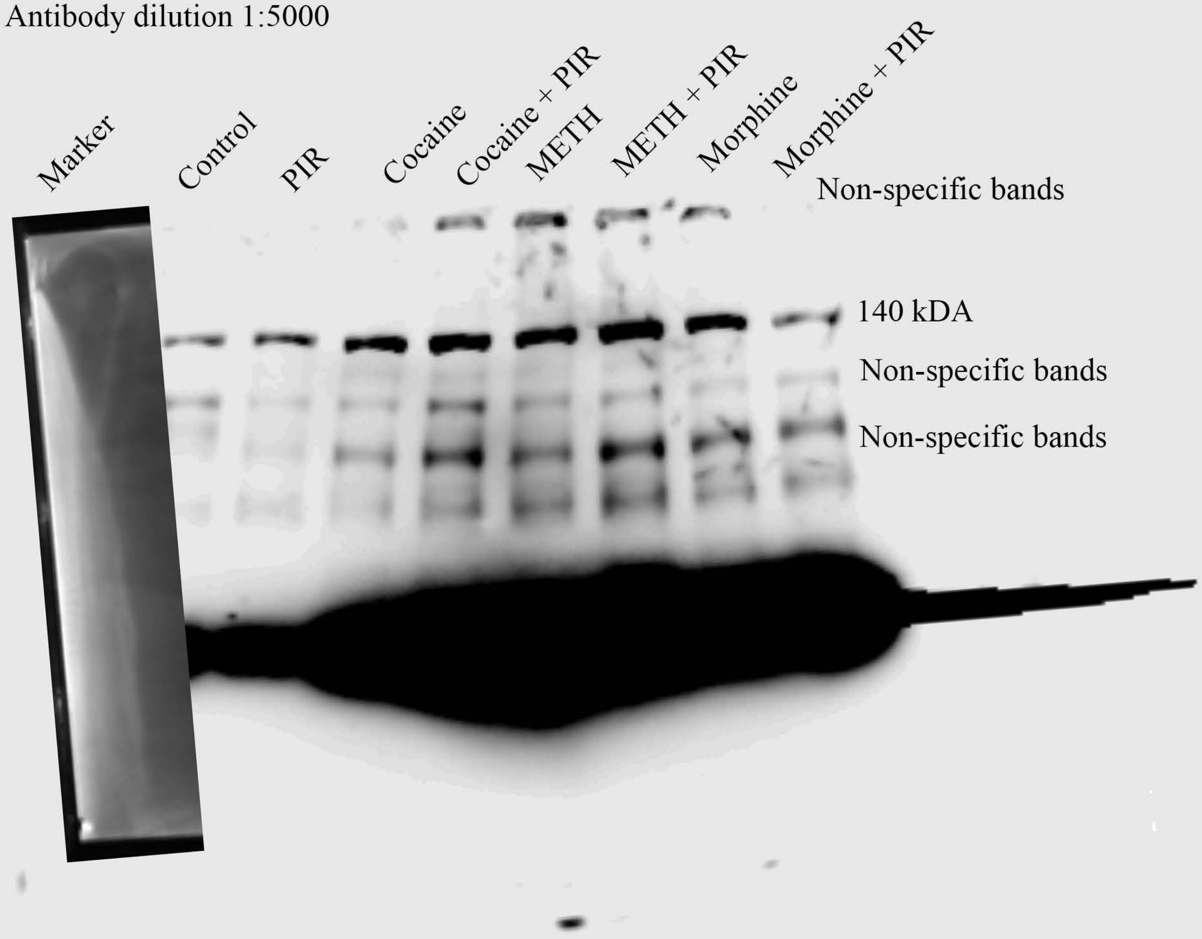

GAPDH for HDAC 4
Human Primary Astrocyte Sample		Primary Antibody dilution 1:1000
Fig. 2 A		Secondary Antibody dilution 1:5000
Image captured by C300 azure biosystems
BIORAD: Clarity Western ECL Substrate

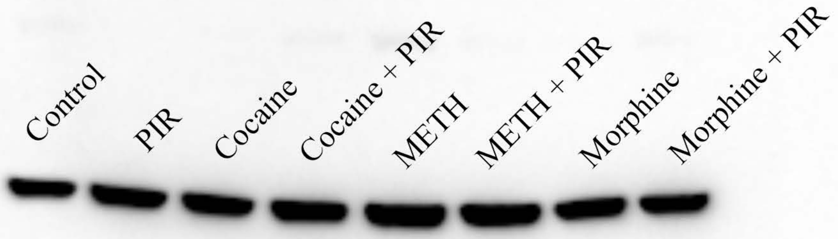

HDAC 5
Human Primary Astrocyte Sample Primary Antibody dilution 1:1000
Fig. 2 B Secondary Antibody dilution 1:5000
Image captured by C300 azure biosystems
BIORAD: Clarity Western ECL Substrate

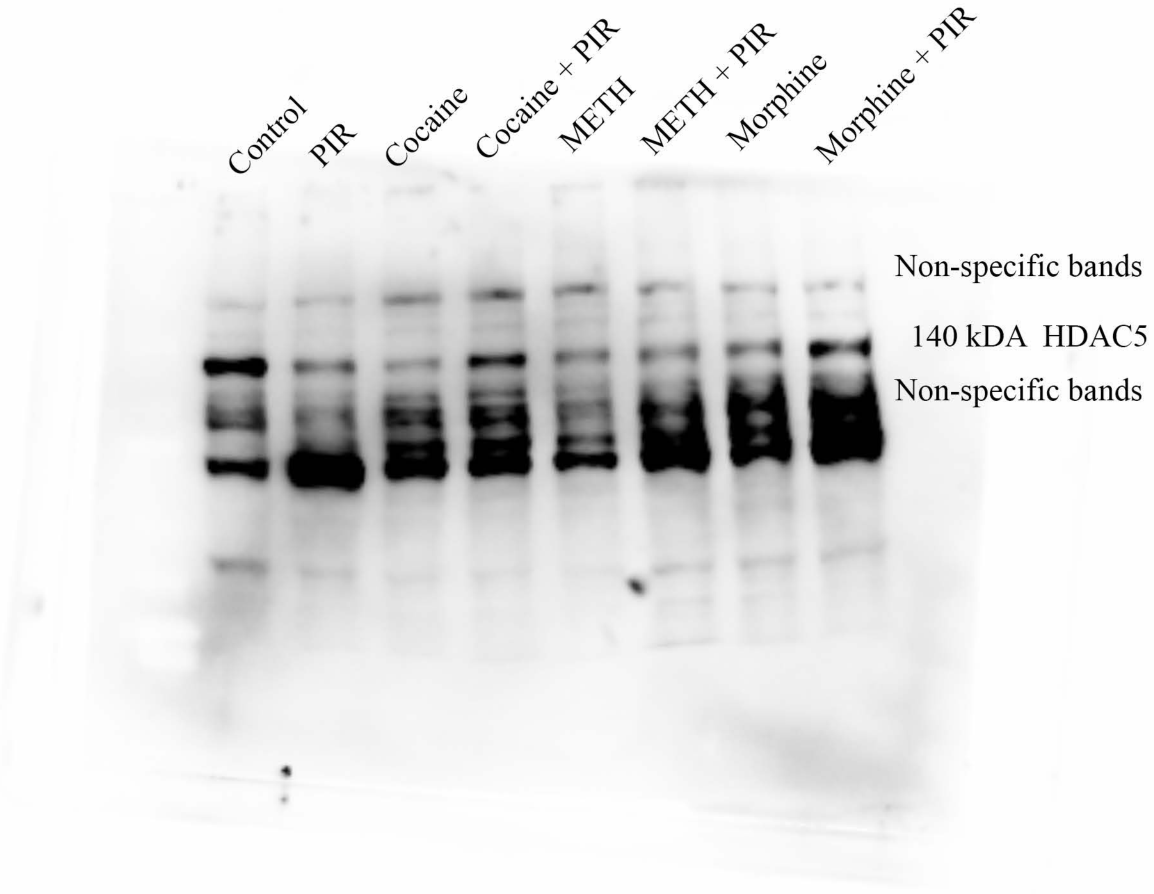

HDAC5 with marker
Human Primary Astrocyte Sample
Fig. 2 B
Image captured by C300 azure biosystems
BIORAD: Clarity Western ECL Substrate

Primary Antibody dilution 1:1000
Secondary Antibody dilution 1:5000

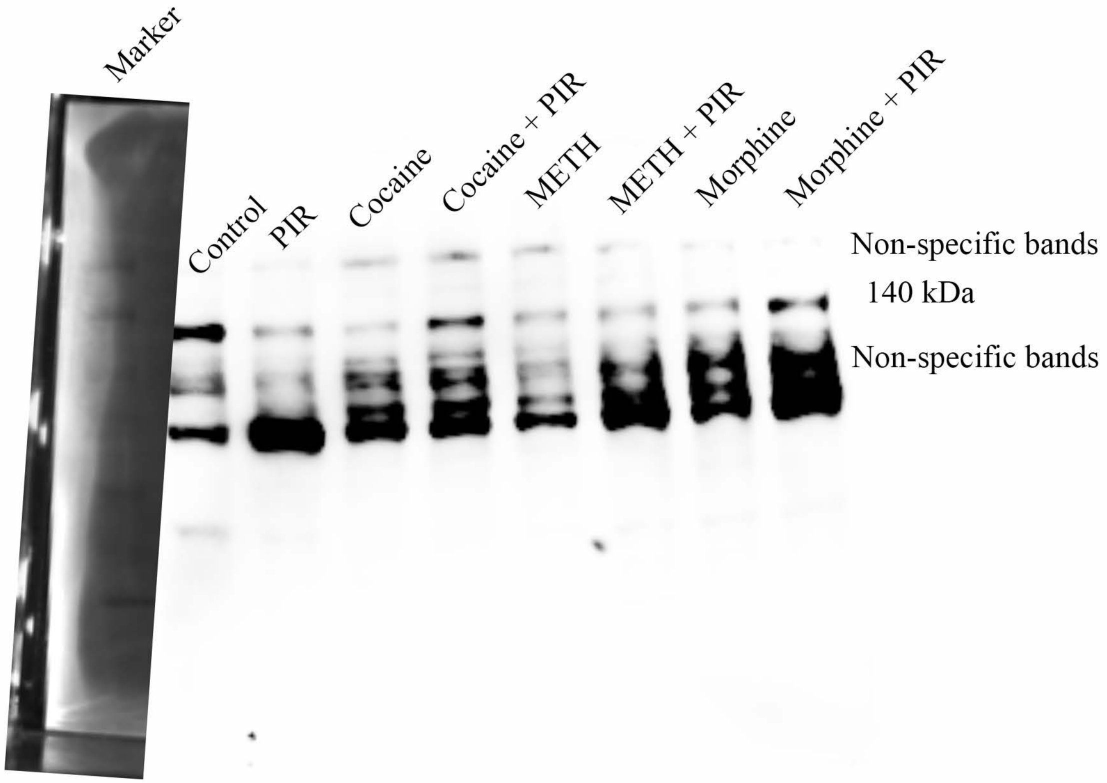

GAPDH for HDAC 5
Human Primary Astrocyte Sample Primary Antibody dilution 1:1000
Fig. 2 B Secondary Antibody dilution 1:5000
Image captured by C300 azure biosystems
BIORAD: Clarity Western ECL Substrate

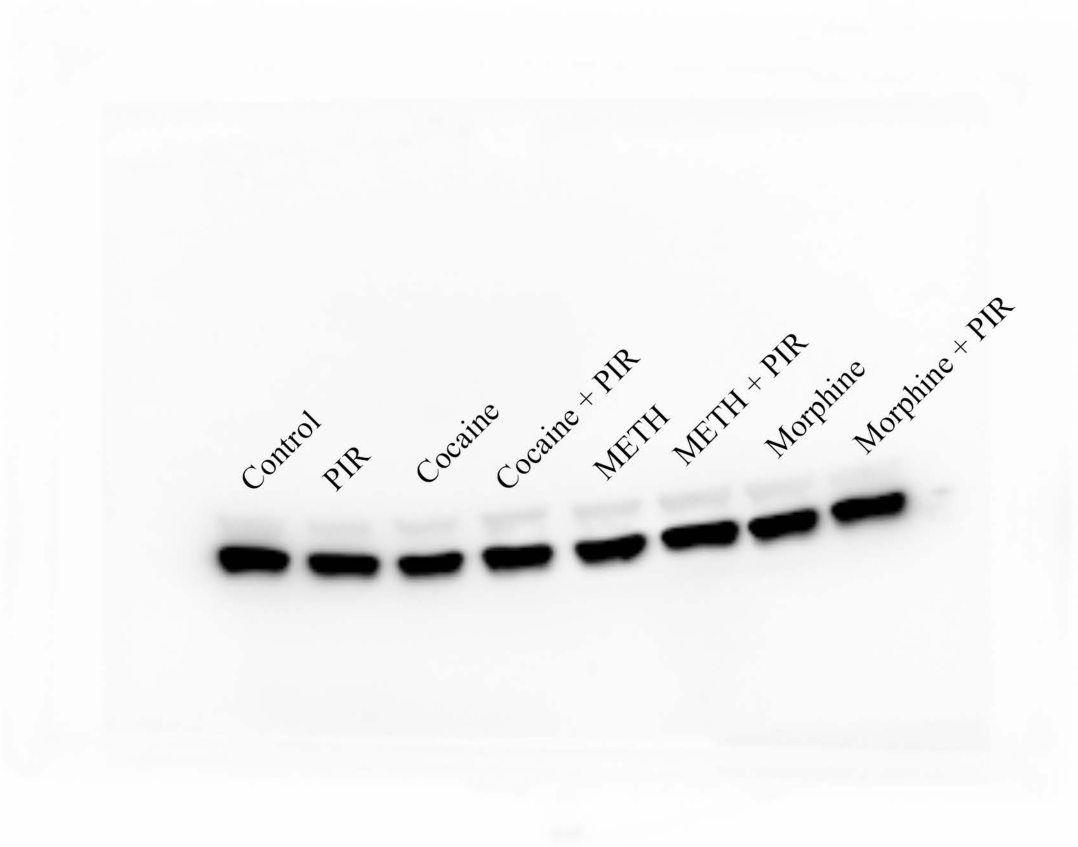

HDAC 6

Human Primary Astrocyte Sample | Primary Antibody dilution 1:1000

Fig. 2 C | Secondary Antibody dilution 1:5000

Image captured by C300 azure biosystems

BIORAD: Clarity Western ECL Substrate

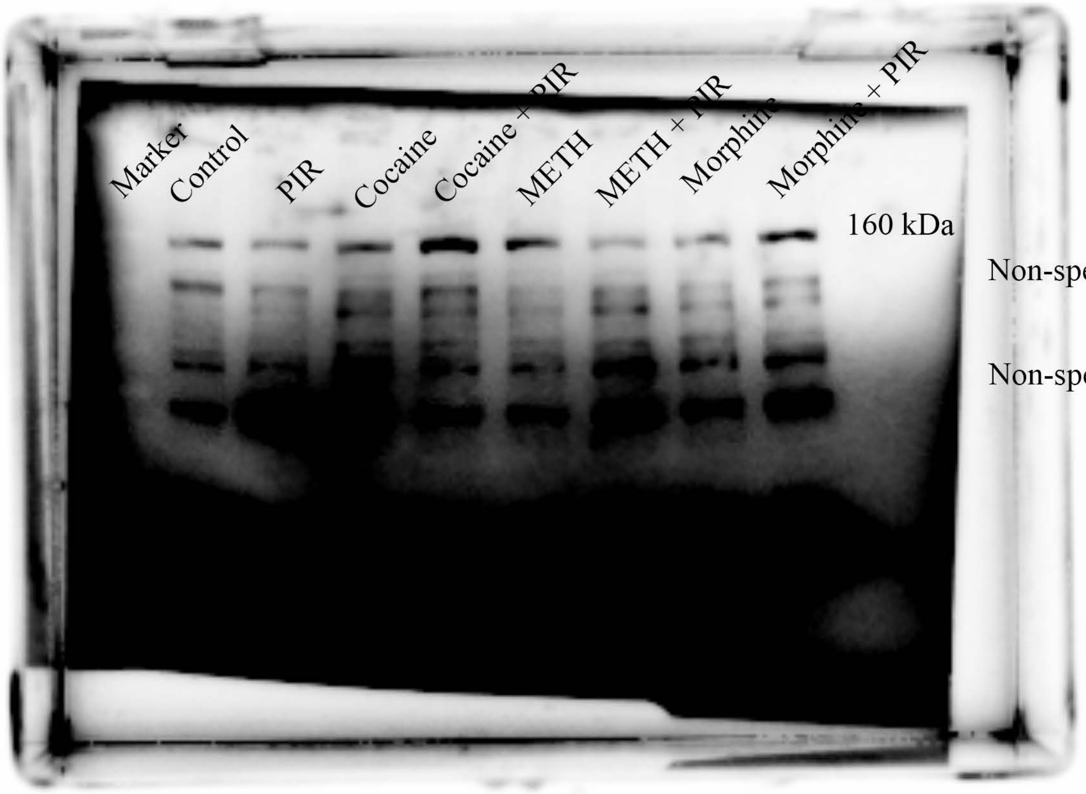

HDAC6 with marker
Human Primary Astrocyte Sample
Fig. 2 C
Image captured by C300 azure biosystems
BIORAD: Clarity Western ECL Substrate

Primary Antibody dilution 1:1000
Secondary Antibody dilution 1:5000

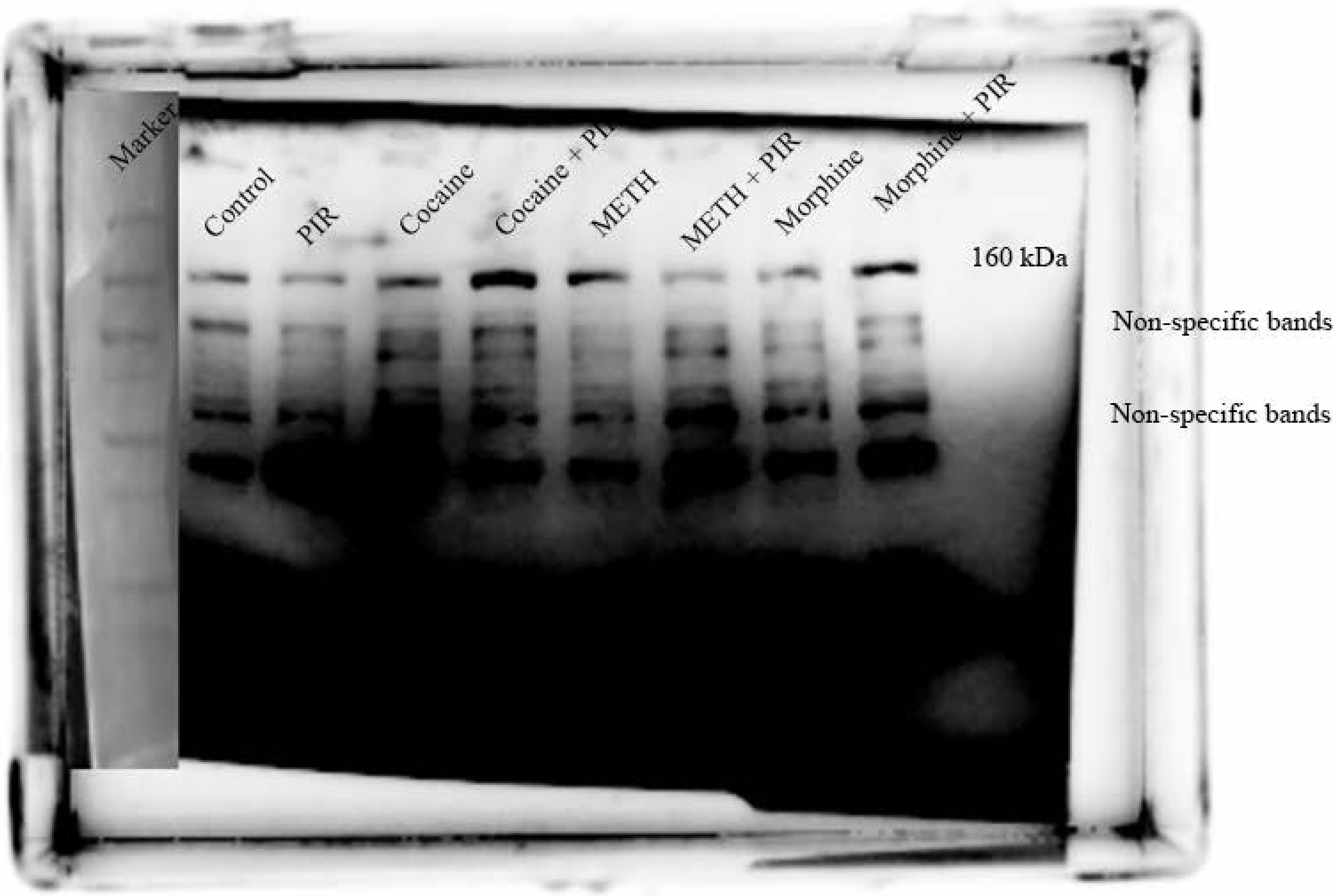

GAPDH for HDAC 6
Human Primary Astrocyte Sample   Primary Antibody dilution 1:1000
Fig. 2 C   Secondary Antibody dilution 1:5000
Image captured by C300 azure biosystems
BIORAD: Clarity Western ECL Substrate

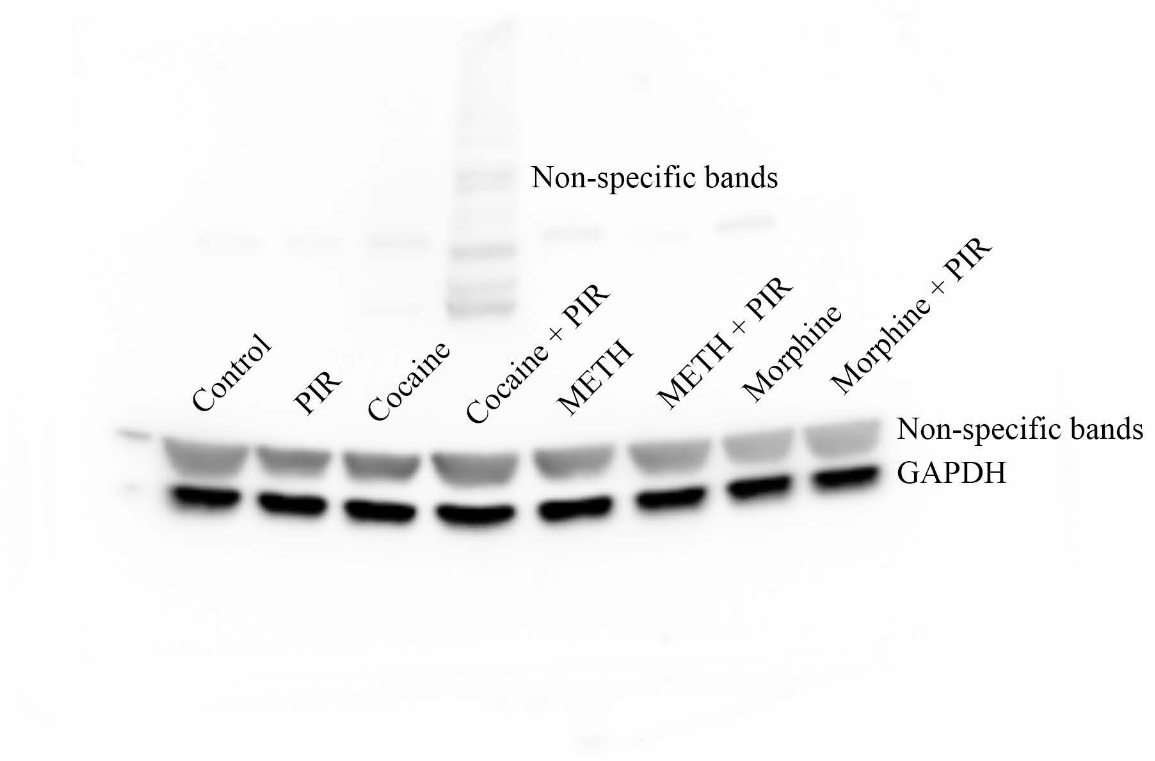

HDAC 7
Human Primary Astrocyte Sample   Primary Antibody dilution 1:1000
Fig. 2 D   Secondary Antibody dilution 1:5000
Image captured by C300 azure biosystems
BIORAD: Clarity Western ECL Substrate

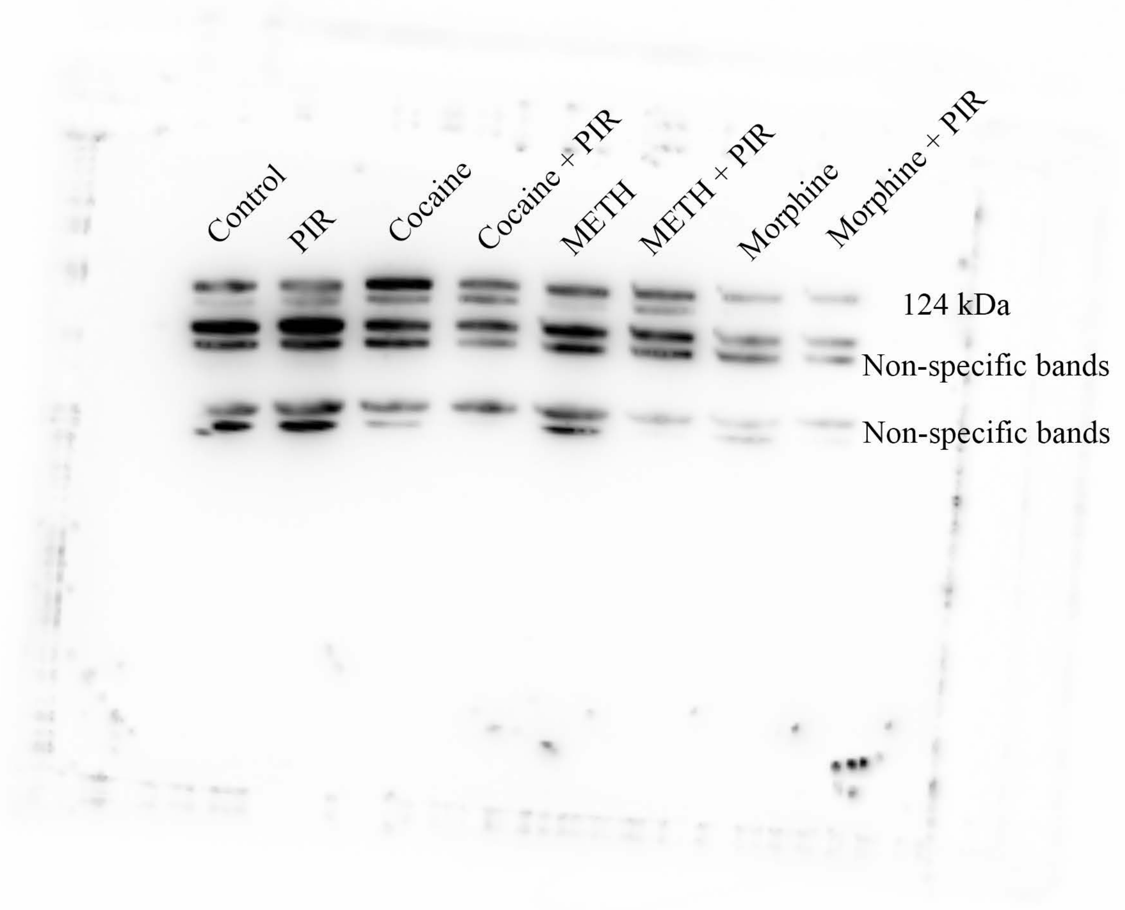

HDAC 7 with marker
Human Primary Astrocyte Sample		Primary Antibody dilution 1:1000
Fig. 2 D						Secondary Antibody dilution 1:5000
Image captured by C300 azure biosystems
BIORAD: Clarity Western ECL Substrate

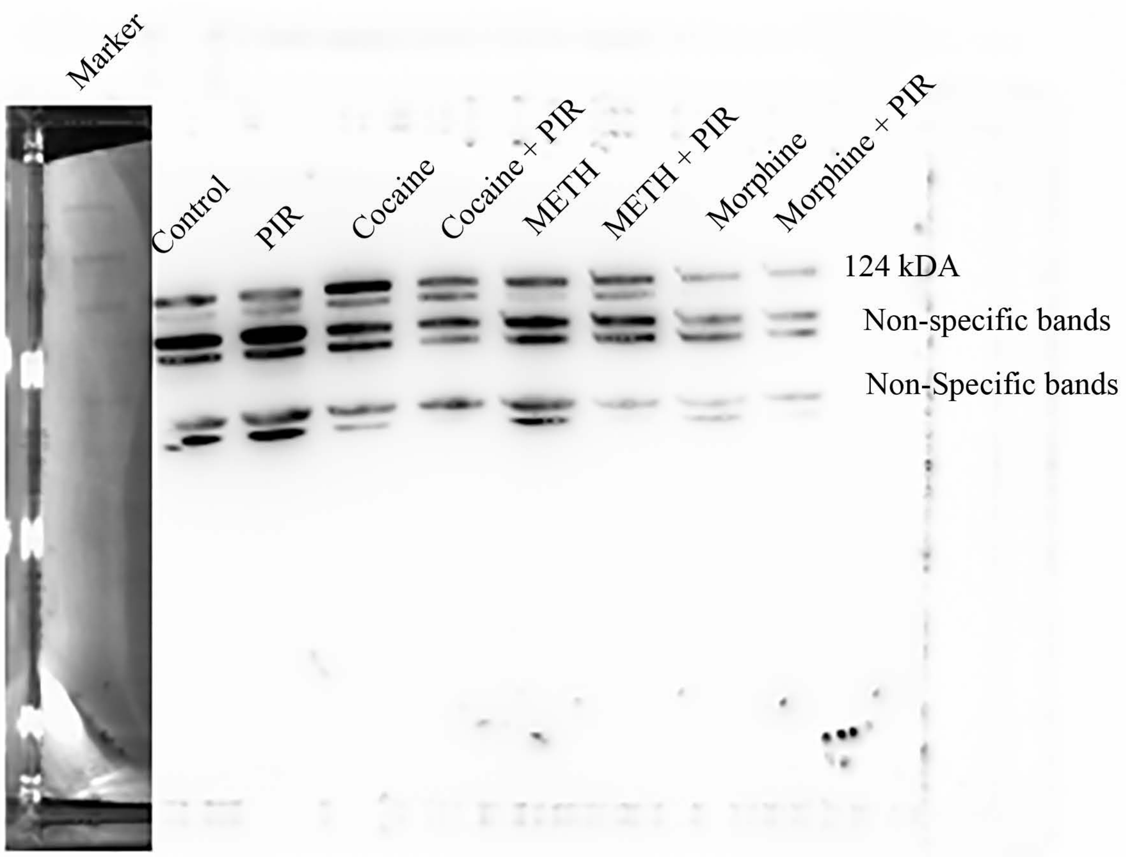

GAPDH for HDAC 7

Human Primary Astrocyte Sample | Primary Antibody dilution 1:1000

Fig. 2 D | Secondary Antibody dilution 1:5000

Image captured by C300 azure biosystems

BIORAD: Clarity Western ECL Substrate

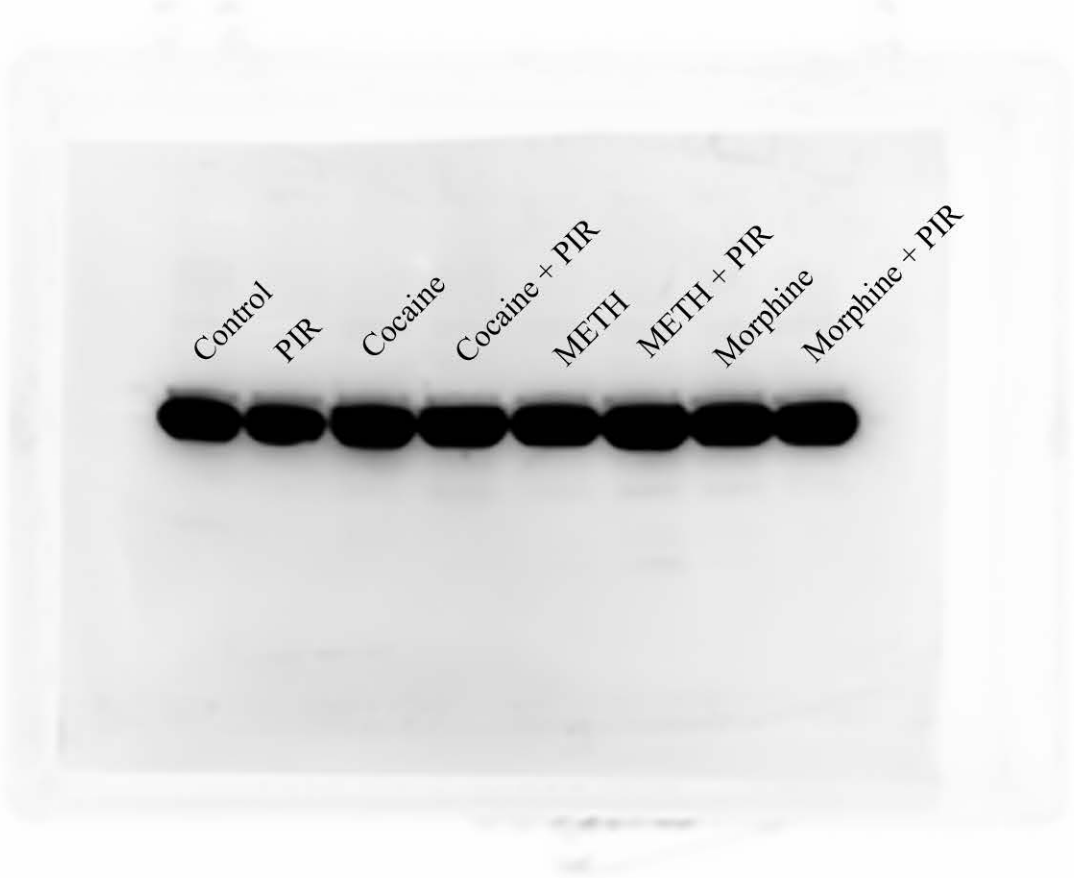

PCAF
Human Primary Astrocyte Sample   Primary Antibody dilution 1:1000
Fig. 3 A   Secondary Antibody dilution 1:5000
Image captured by C300 azure biosystems
BIORAD: Clarity Western ECL Substrate

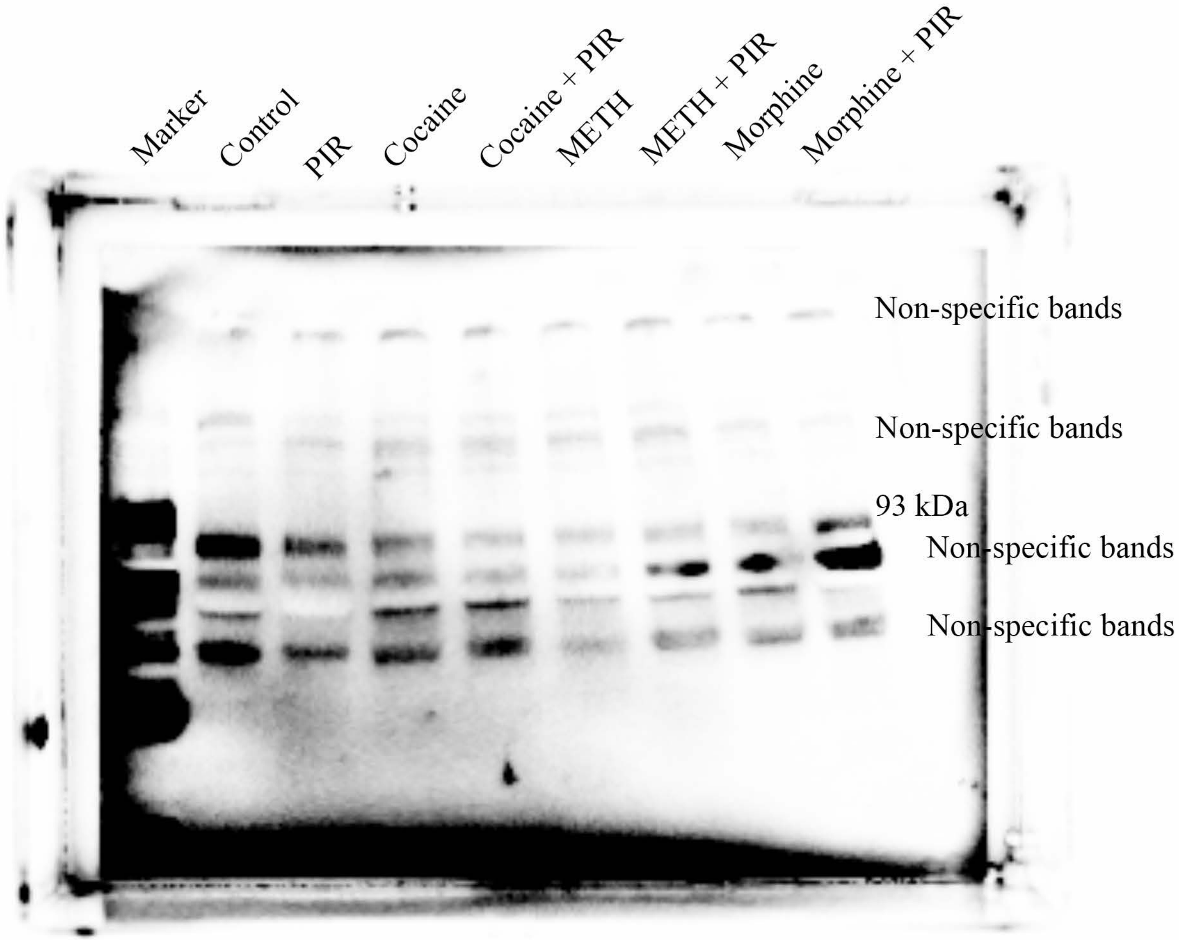

PCAF with marker
Human Primary Astrocyte Sample
Fig. 3 A
Image captured by C300 azure biosystems
BIORAD: Clarity Western ECL Substrate

Primary Antibody dilution 1:1000
Secondary Antibody dilution 1:5000

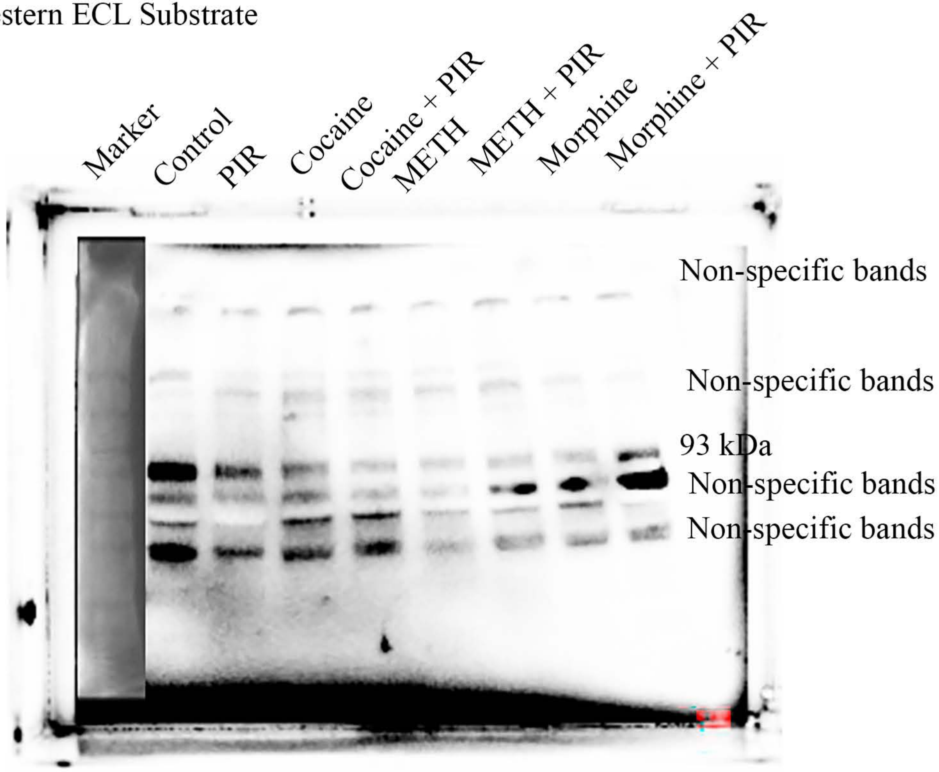

GAPDH for PCAF

Human Primary Astrocyte Sample   Primary Antibody dilution 1:1000

Fig. 3 A   Secondary Antibody dilution 1:5000

Image captured by C300 azure biosystems

BIORAD: Clarity Western ECL Substrate

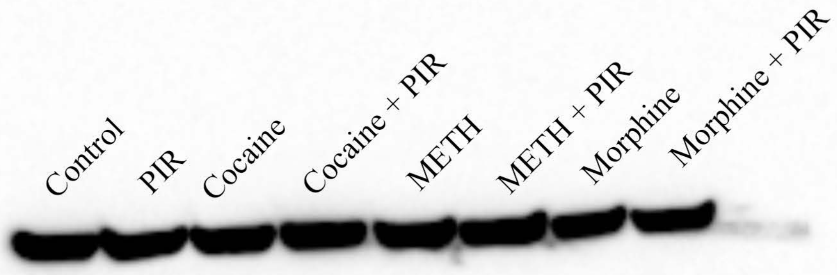

P300

Human Primary Astrocyte Sample

Fig. 3 B

Image captured by C300 azure biosystems

BIORAD: Clarity Western ECL Substrate

Primary Antibody dilution 1:1000

Secondary Antibody dilution 1:5000

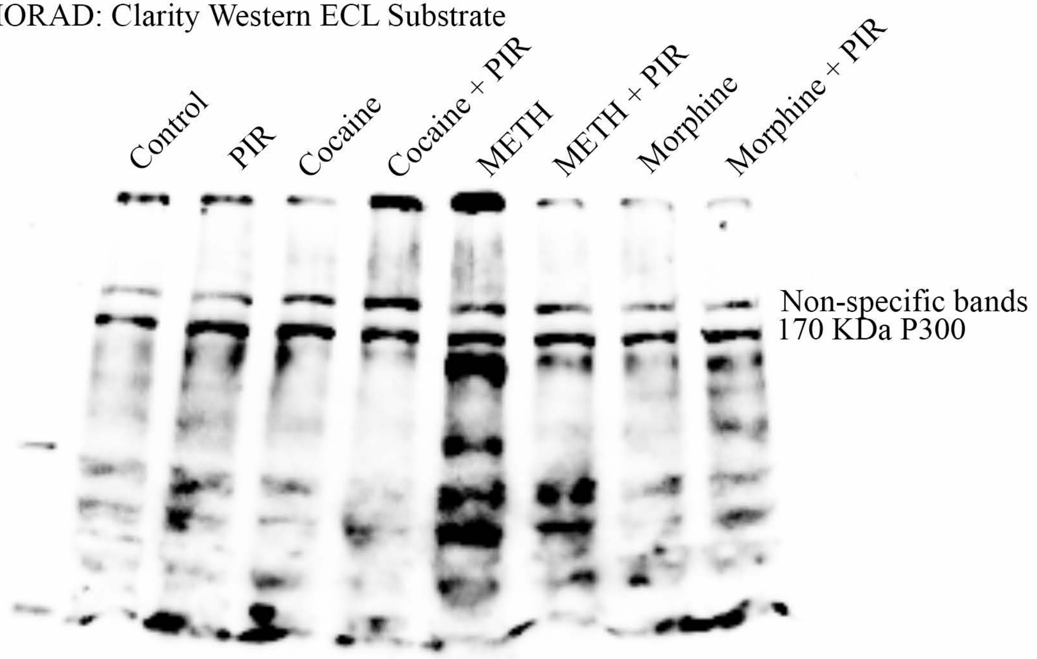

P300 with marker
Human Primary Astrocyte Sample
Primary Antibody dilution 1:1000
Fig. 3 B
Secondary Antibody dilution 1:5000
Image captured by C300 azure biosystems
BIORAD: Clarity Western ECL Substrate

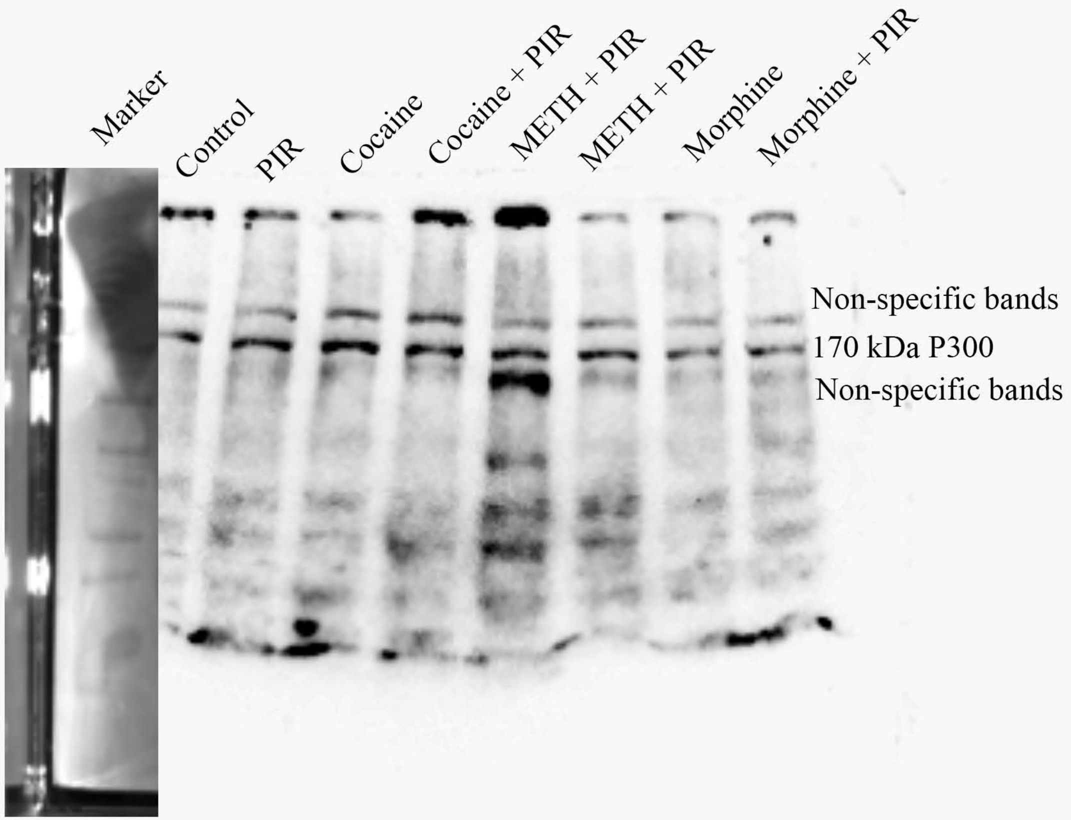

GAPDH for P300

Human Primary Astrocyte Sample | Primary Antibody dilution 1:1000

Fig. 3 B | Secondary Antibody dilution 1:5000

Image captured by C300 azure biosystems

BIORAD: Clarity Western ECL Substrate

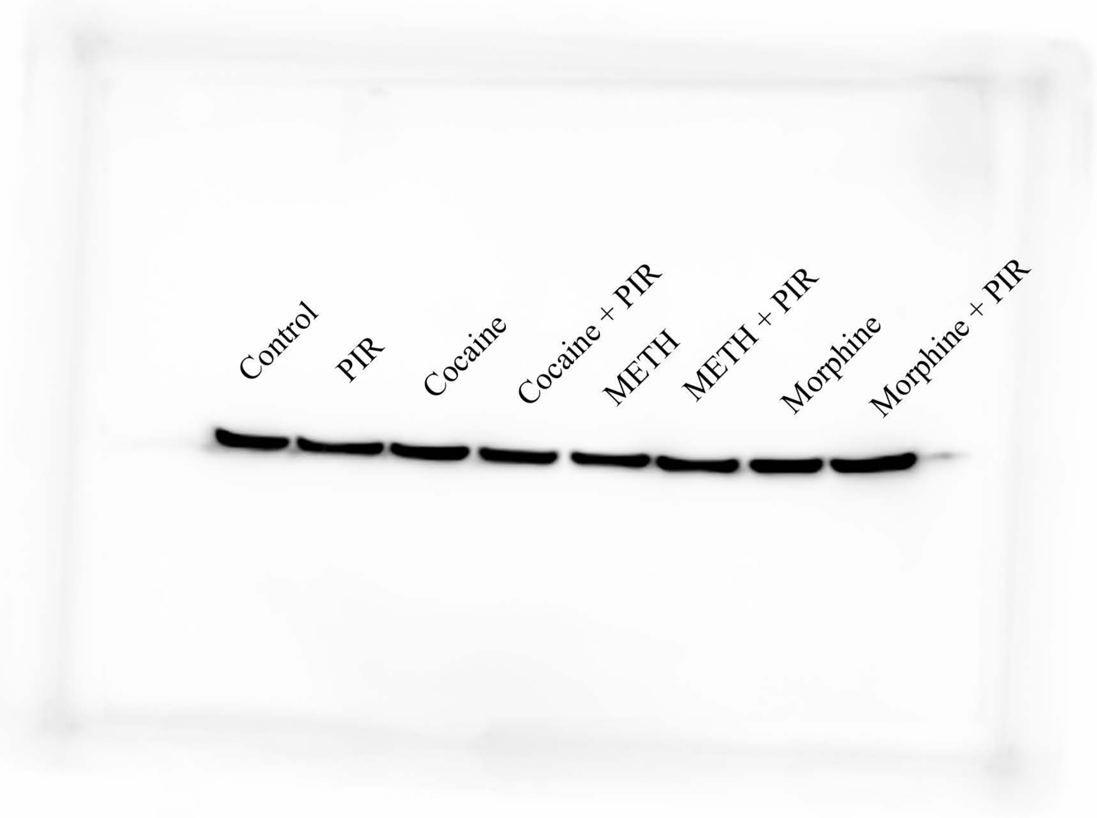

GCN 5
Human Primary Astrocyte Sample		Primary Antibody dilution 1:1000
Fig. 3 C		Secondary Antibody dilution 1:5000
Image captured by C300 azure biosystems
BIORAD: Clarity Western ECL Substrate

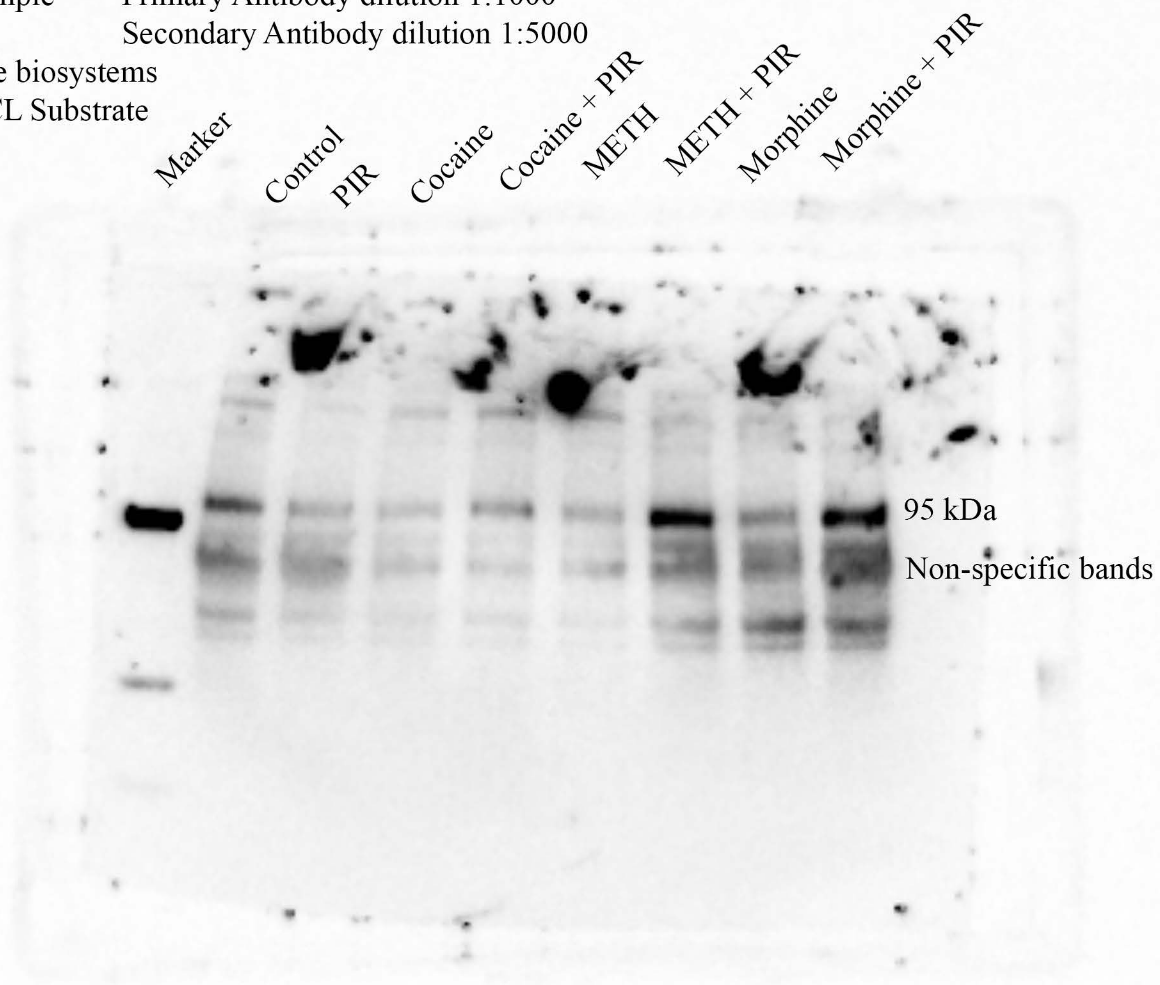

GCN5 with marker
Human Primary Astrocyte Sample
Fig. 3 C
Image captured by C300 azure biosystems
BIORAD: Clarity Western ECL Substrate

Primary Antibody dilution 1:1000
Secondary Antibody dilution 1:5000

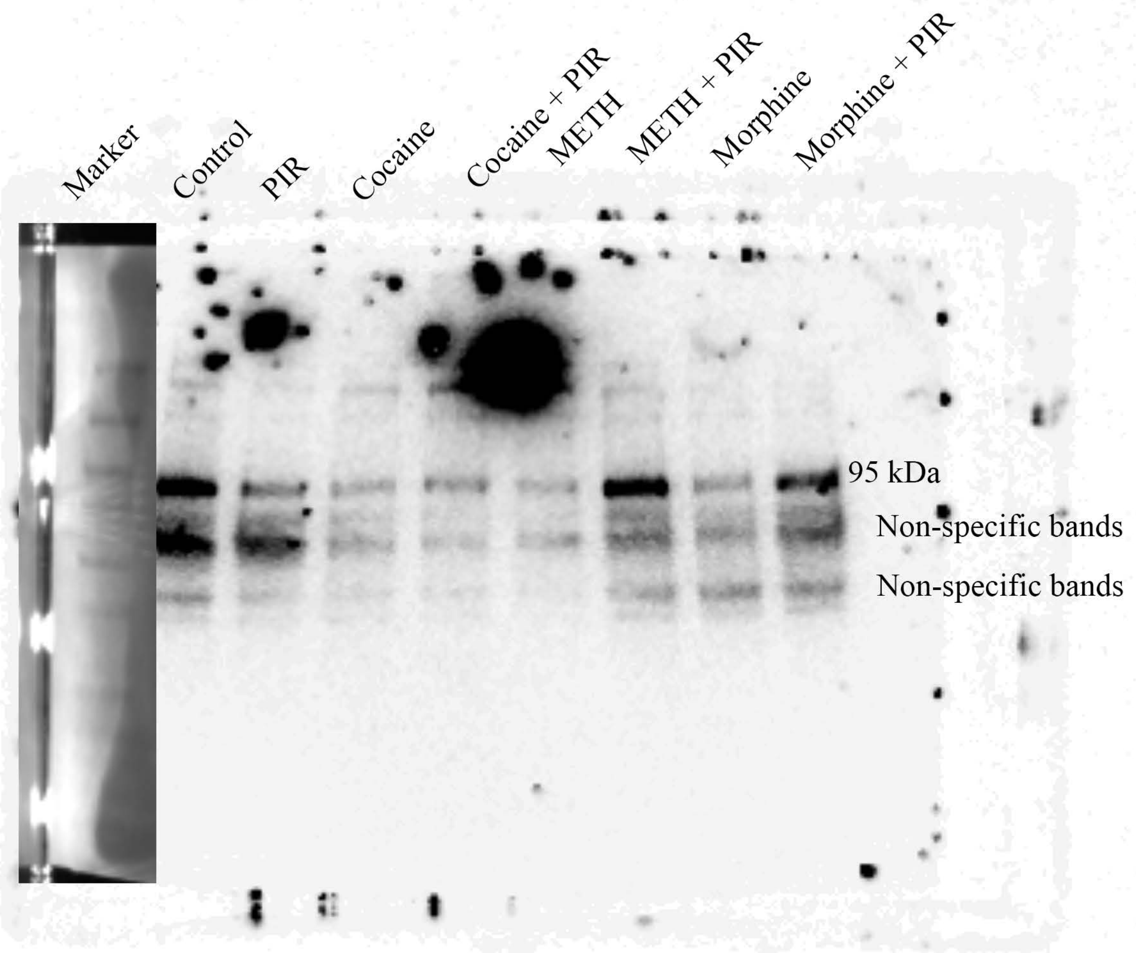

GAPDH for GCN5
Human Primary Astrocyte Sample Primary Antibody dilution 1:1000
Fig. 3 C Secondary Antibody dilution 1:5000
Image captured by C300 azure biosystems
BIORAD: Clarity Western ECL Substrate

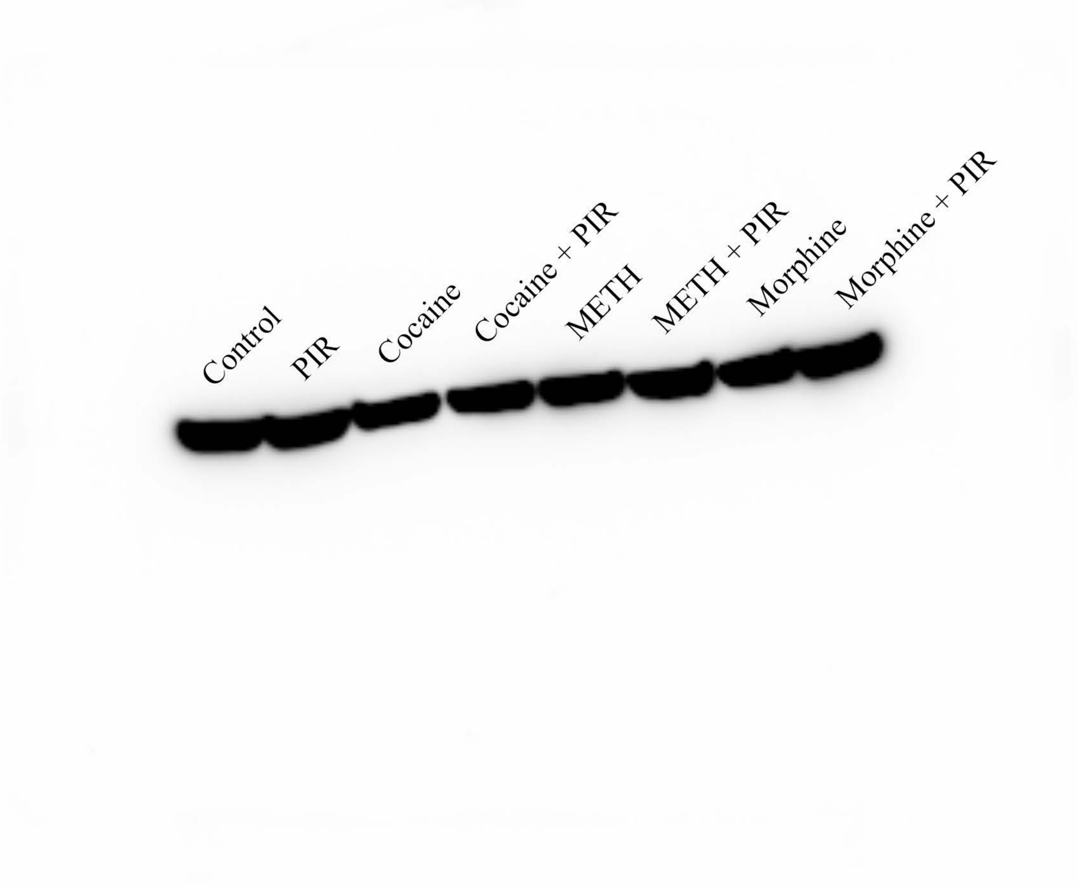

H3K9AC
Human Primary Astrocyte Sample	Primary Antibody dilution 1:1000
Fig. 4 A	Secondary Antibody dilution 1:5000
Image captured by C300 azure biosystems
BIORAD: Clarity Western ECL Substrate

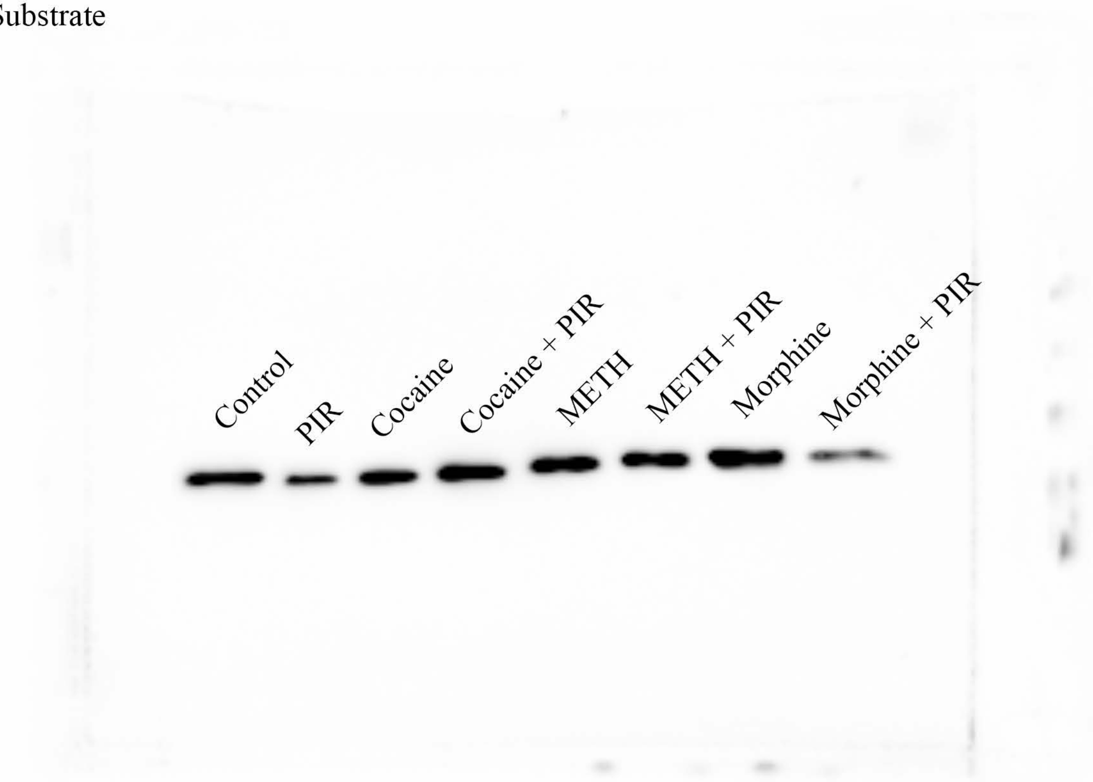

H3K9AC with Marker

Human Primary Astrocyte Sample

Primary Antibody dilution 1:1000

Fig. 4 A

Secondary Antibody dilution 1:5000

Image captured by C300 azure biosystems

BIORAD: Clarity Western ECL Substrate

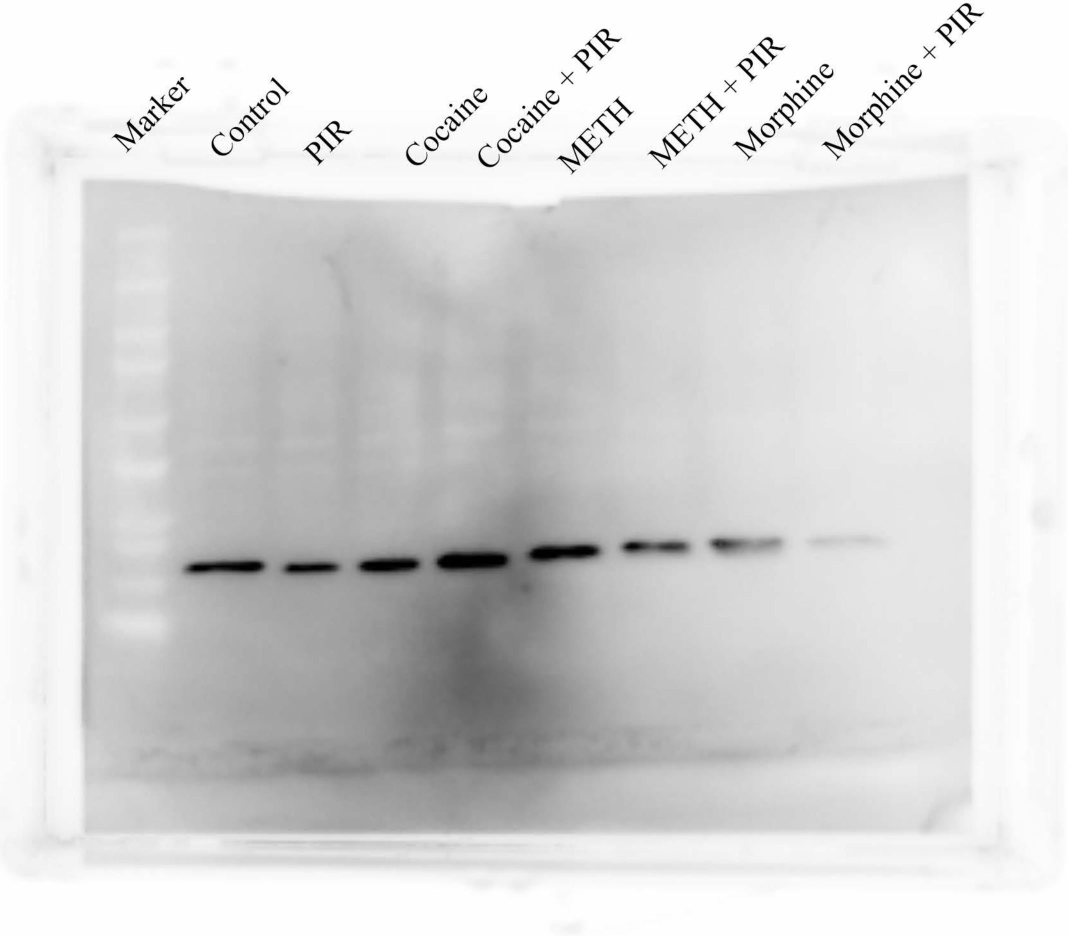

H3K14AC
Human Primary Astrocyte Sample
Primary Antibody dilution 1:1000
Fig. 4 B
Secondary Antibody dilution 1:5000
Image captured by C300 azure biosystems
BIORAD: Clarity Western ECL Substrate

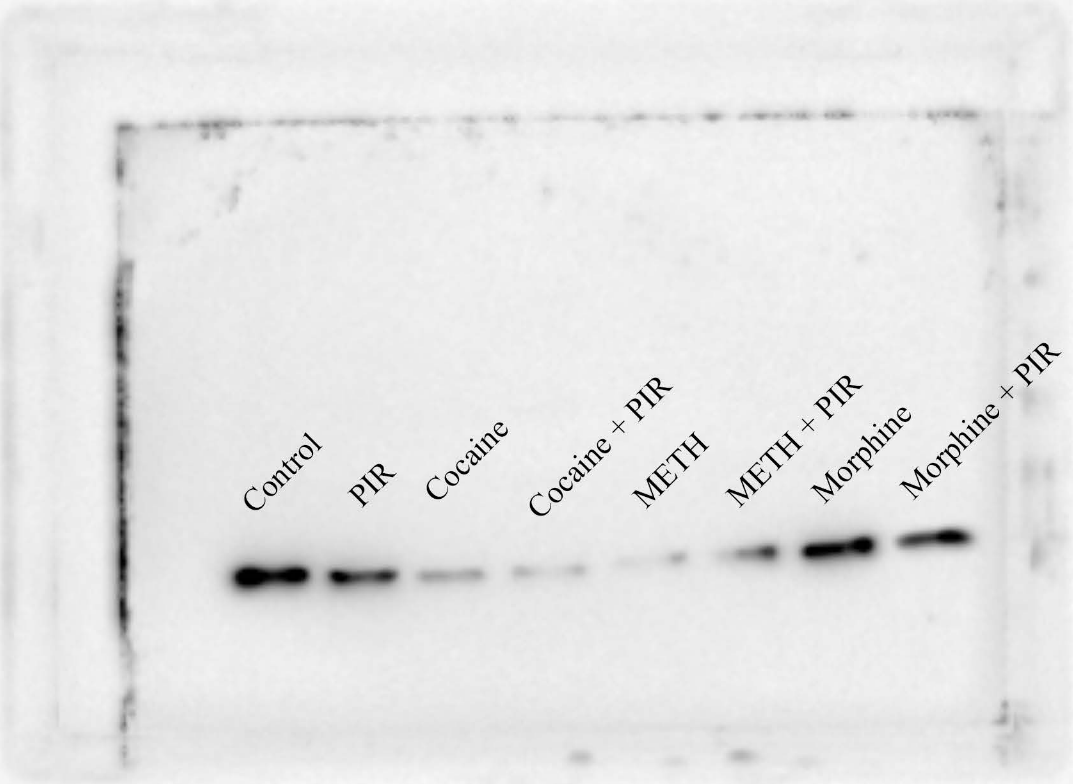

H3K14AC with Marker
Human Primary Astrocyte Sample
Fig. 4 B
Image captured by C300 azure biosystems
BIORAD: Clarity Western ECL Substrate

Primary Antibody dilution 1:1000
Secondary Antibody dilution 1:5000

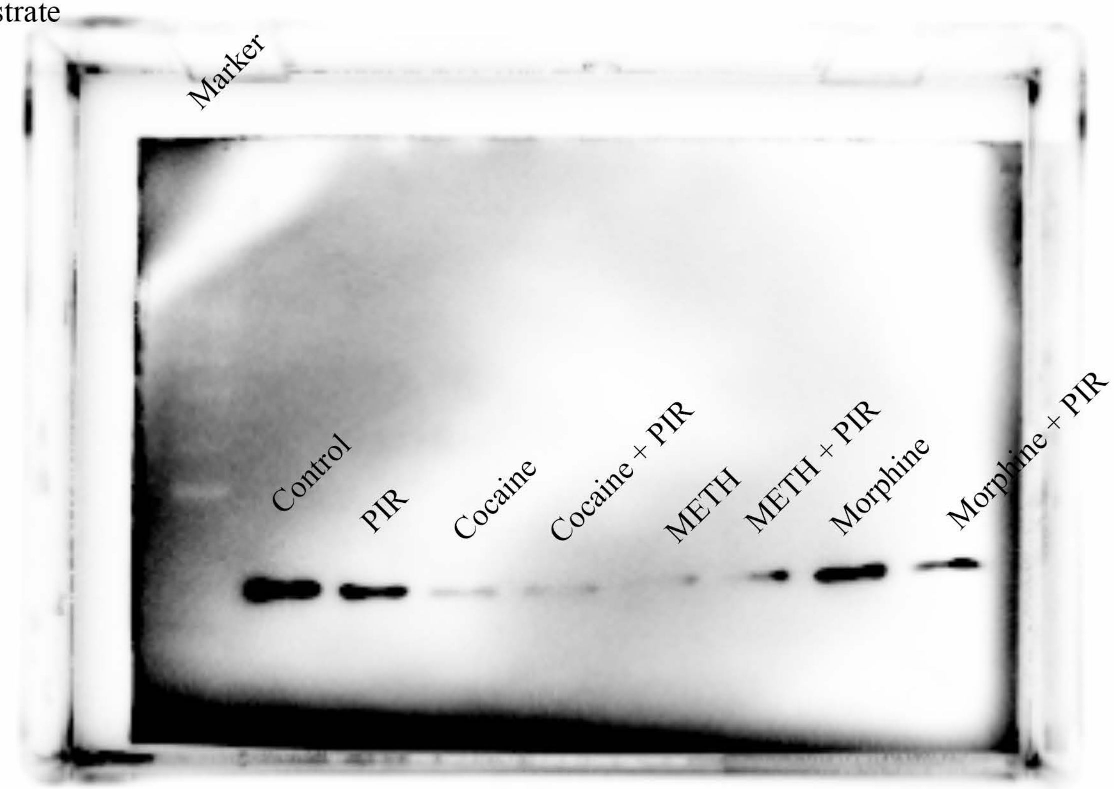

H3K18AC
Human Primary Astrocyte Sample		Primary Antibody dilution 1:1000
Fig. 4 C		Secondary Antibody dilution 1:5000
Image captured by C300 azure biosystems
BIORAD: Clarity Western ECL Substrate

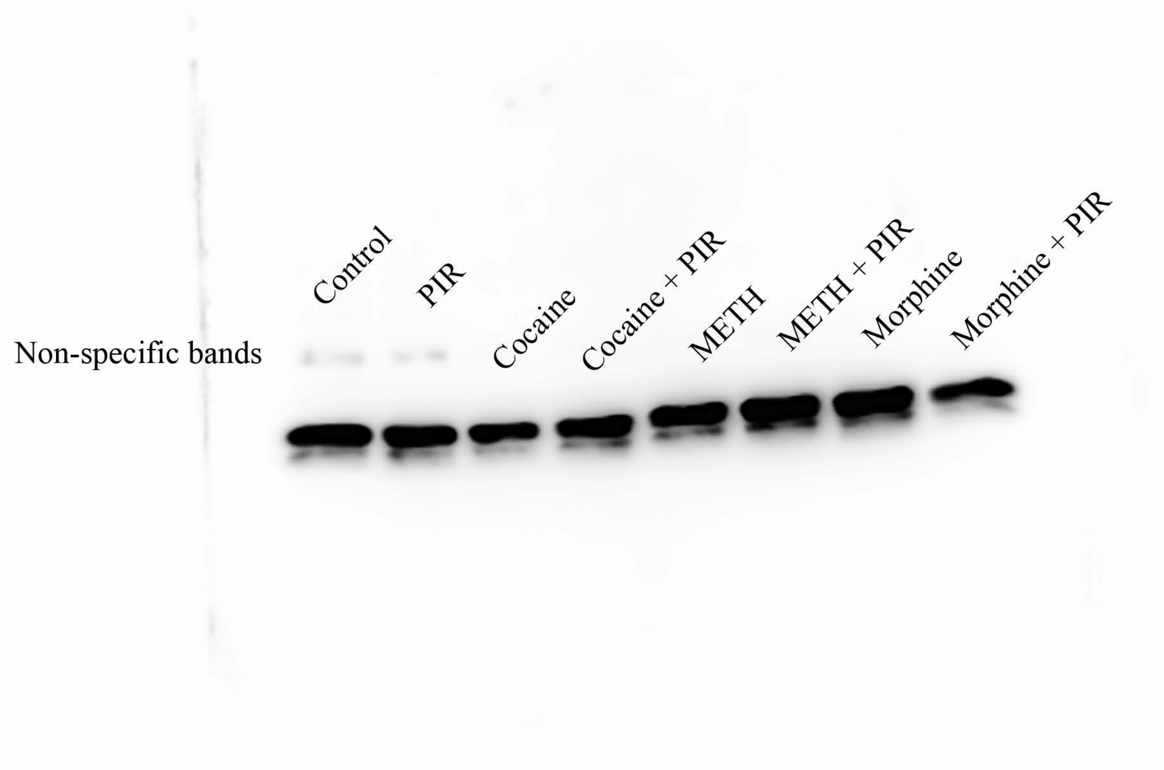

H3k27AC

Human Primary Astrocyte Sample

Fig. 4 D

Image captured by C300 azure biosystems

BIORAD: Clarity Western ECL Substrate

Primary Antibody dilution 1:1000

Secondary Antibody dilution 1:5000

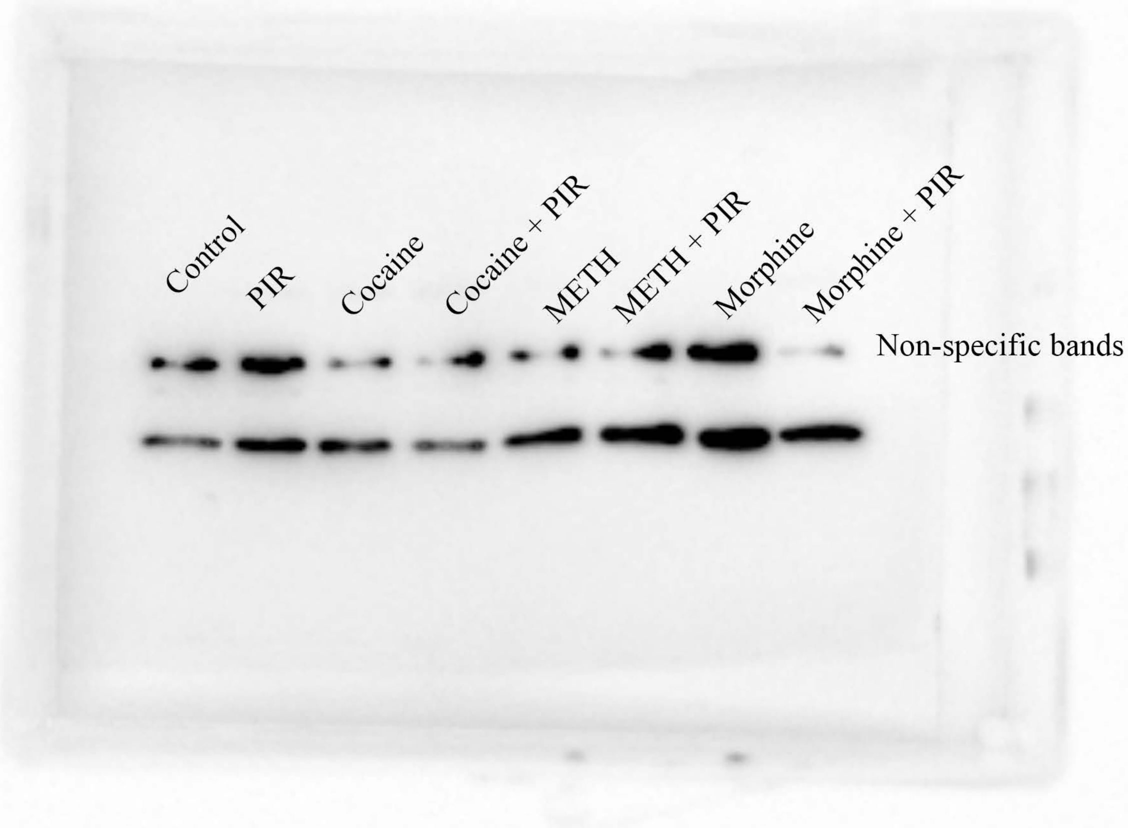

H3k27AC with marker

Human Primary Astrocyte Sample

Fig. 4 D

Image captured by C300 azure biosystems

BIORAD: Clarity Western ECL Substrate

Primary Antibody dilution 1:1000

Secondary Antibody dilution 1:5000

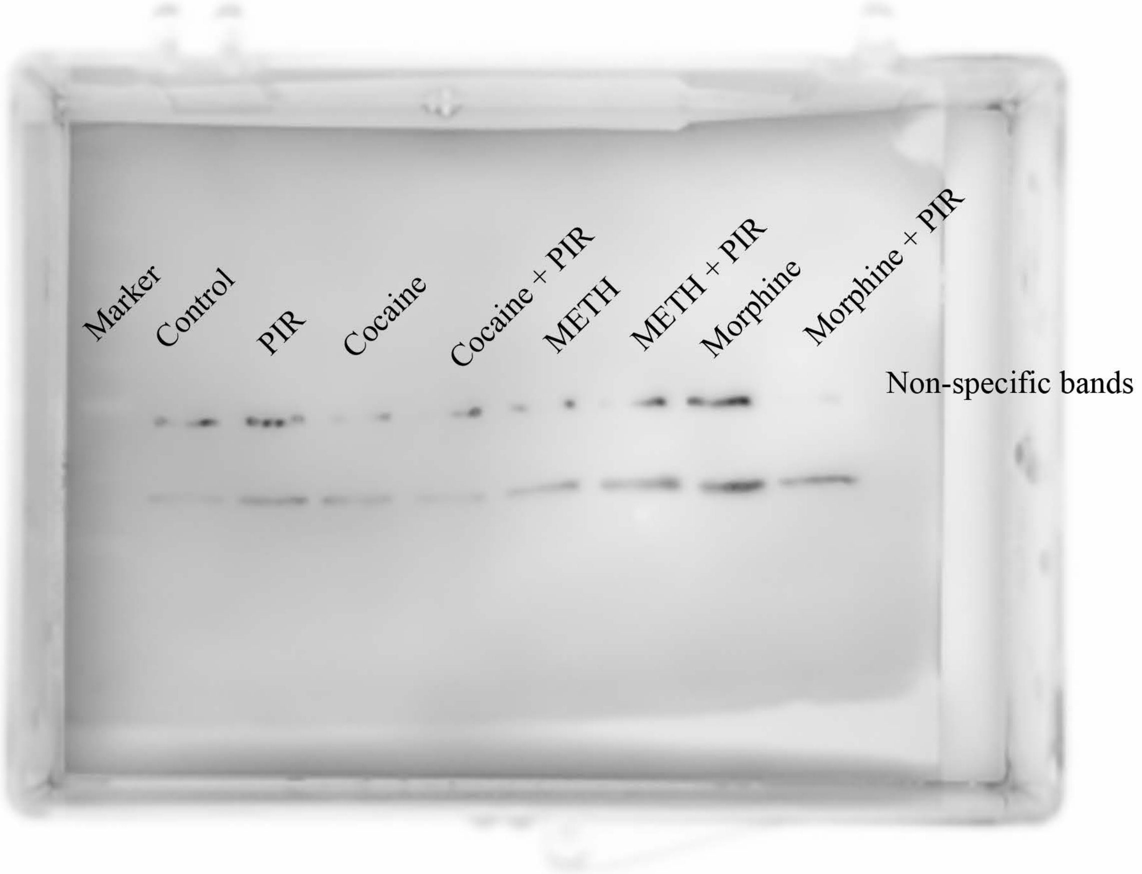

H3K56AC
Human Primary Astrocyte Sample
Fig. 4 E
Image captured by C300 azure biosystems
BIORAD: Clarity Western ECL Substrate

Primary Antibody dilution 1:1000
Secondary Antibody dilution 1:5000

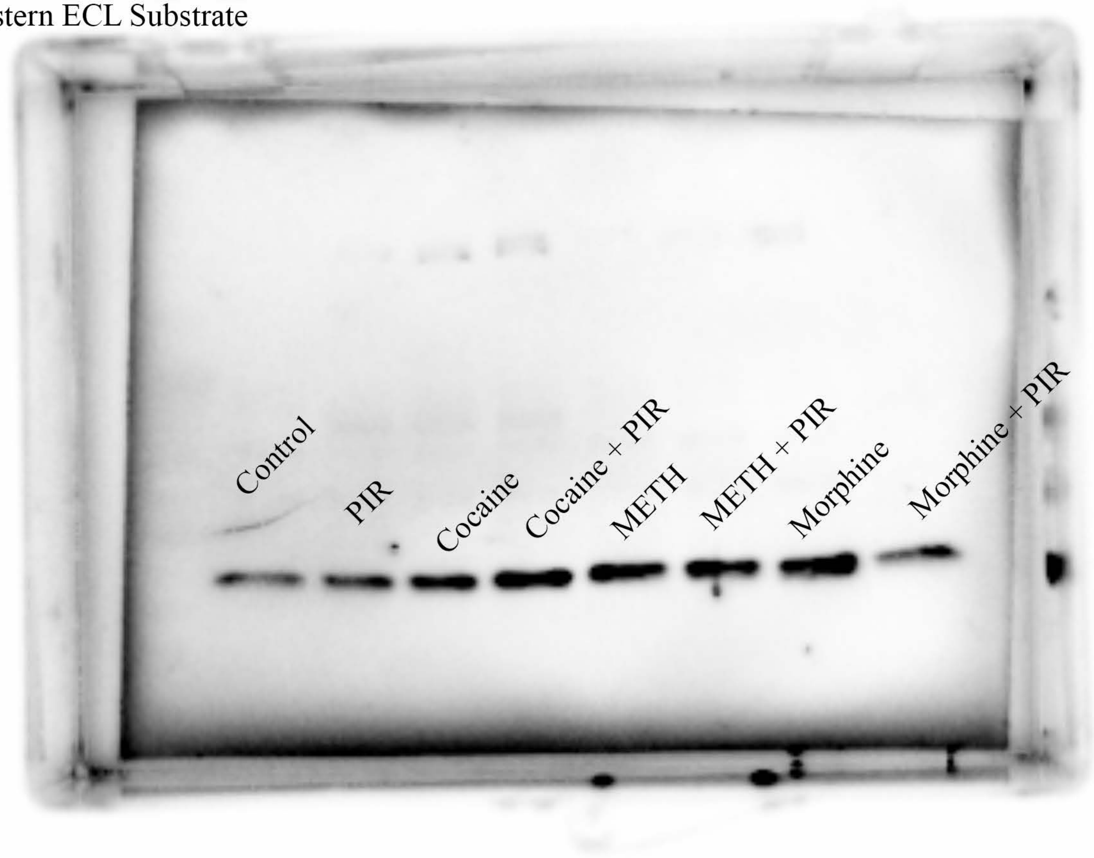

H3K56AC with marker
Human Primary Astrocyte Sample		Primary Antibody dilution 1:1000
Fig. 4 E		Secondary Antibody dilution 1:5000
Image captured by C300 azure biosystems
BIORAD: Clarity Western ECL Substrate

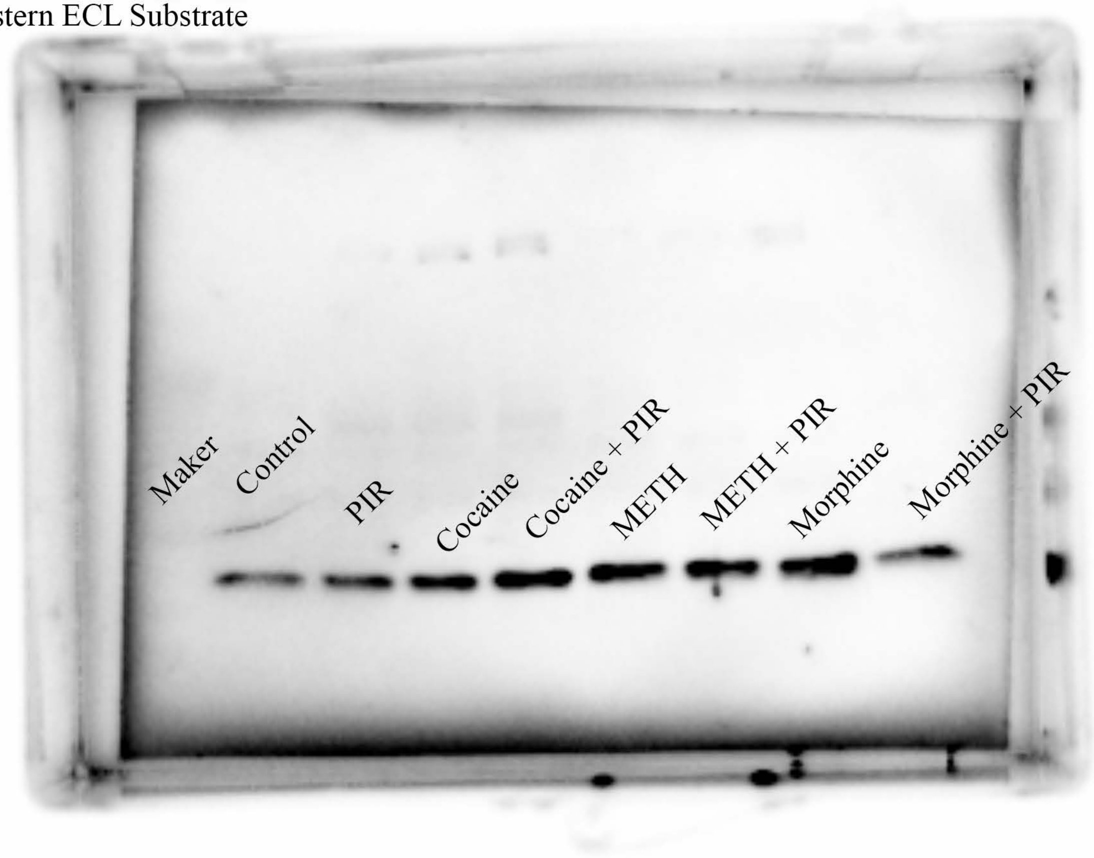

Total H3
Human Primary Astrocyte Sample Primary Antibody dilution 1:1000
Fig. 4 F Secondary Antibody dilution 1:5000
Image captured by C300 azure biosystems
BIORAD: Clarity Western ECL Substrate

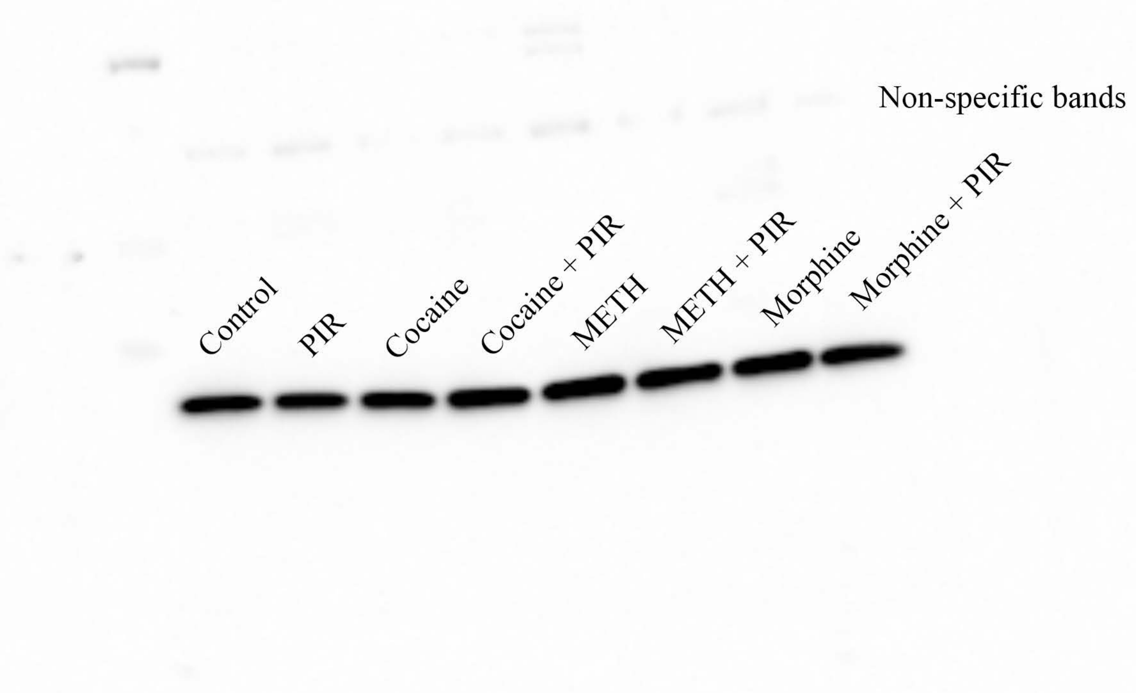

Supplement: S1 Raw images — (PDF) [file pone.0252895.s001.pdf]
