# Supplementary figures and images for: Psychostimulants and opioids differentially influence the epigenetic modification of histone acetyltransferase and histone deacetylase in astrocytes
Source: PLoS One. 2021 Jun 11;16(6):e0252895. doi: 10.1371/journal.pone.0252895 (PMC8195369; doi:10.1371/journal.pone.0252895)

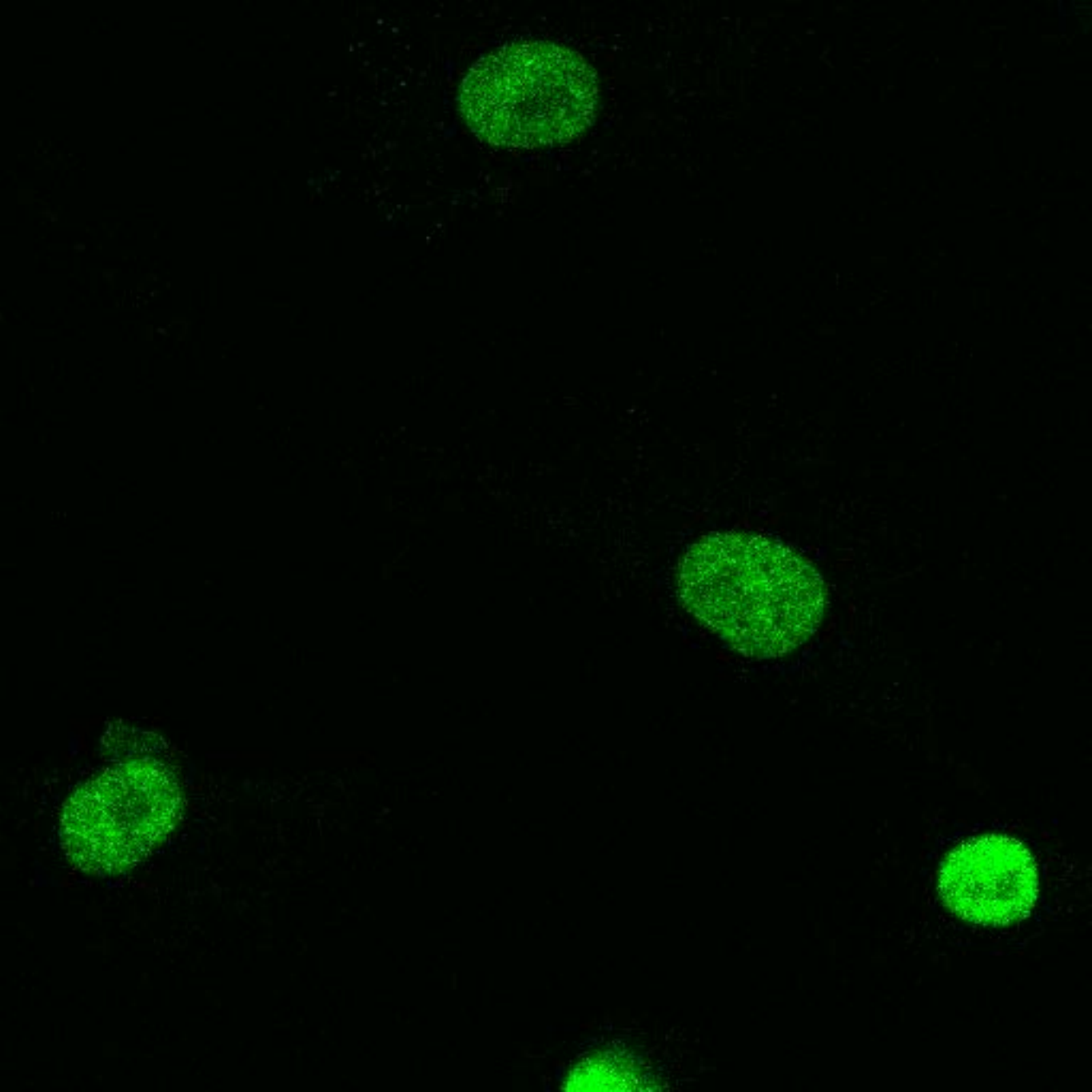

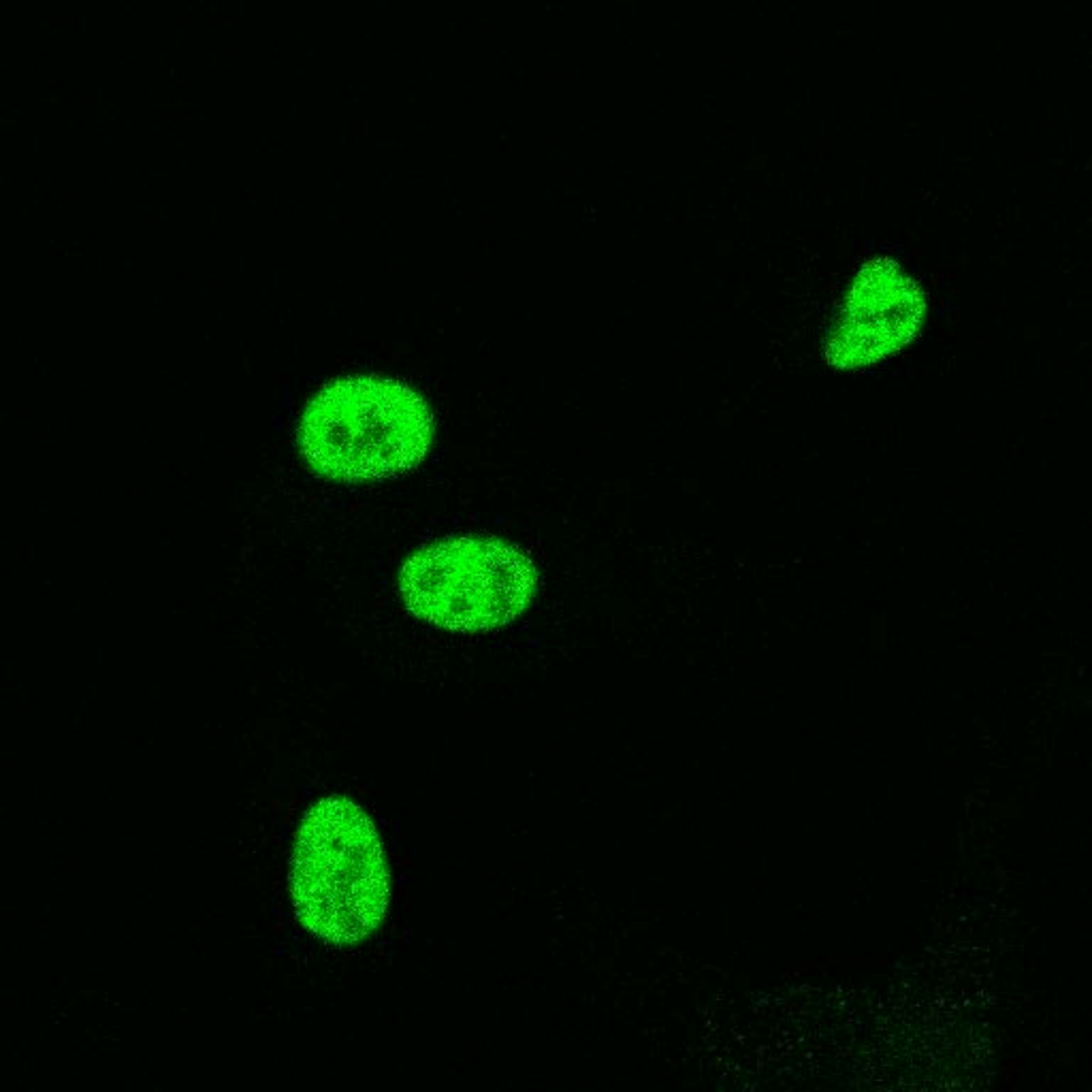

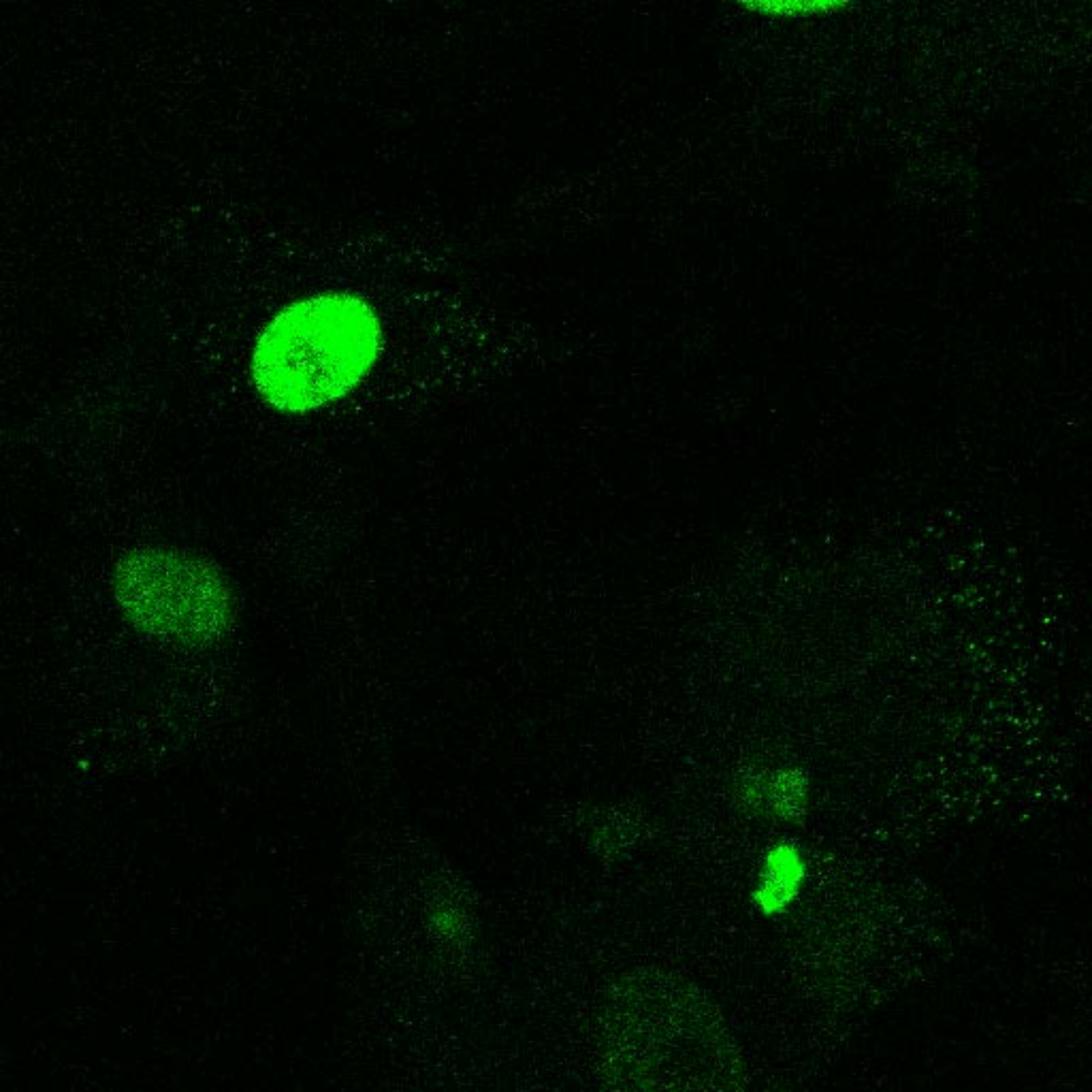

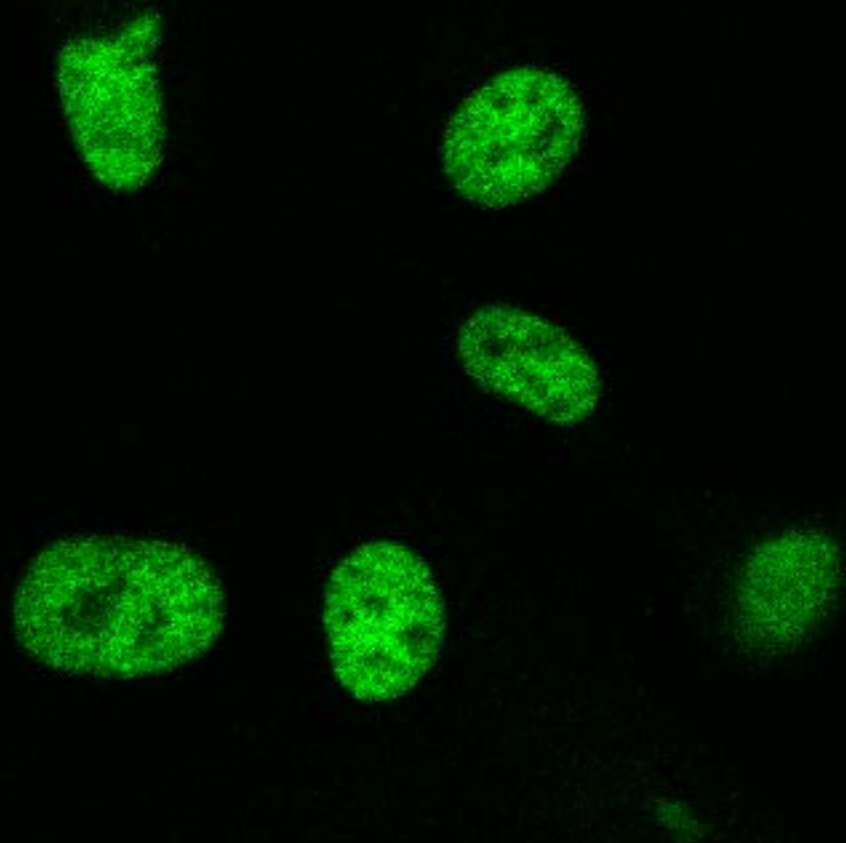

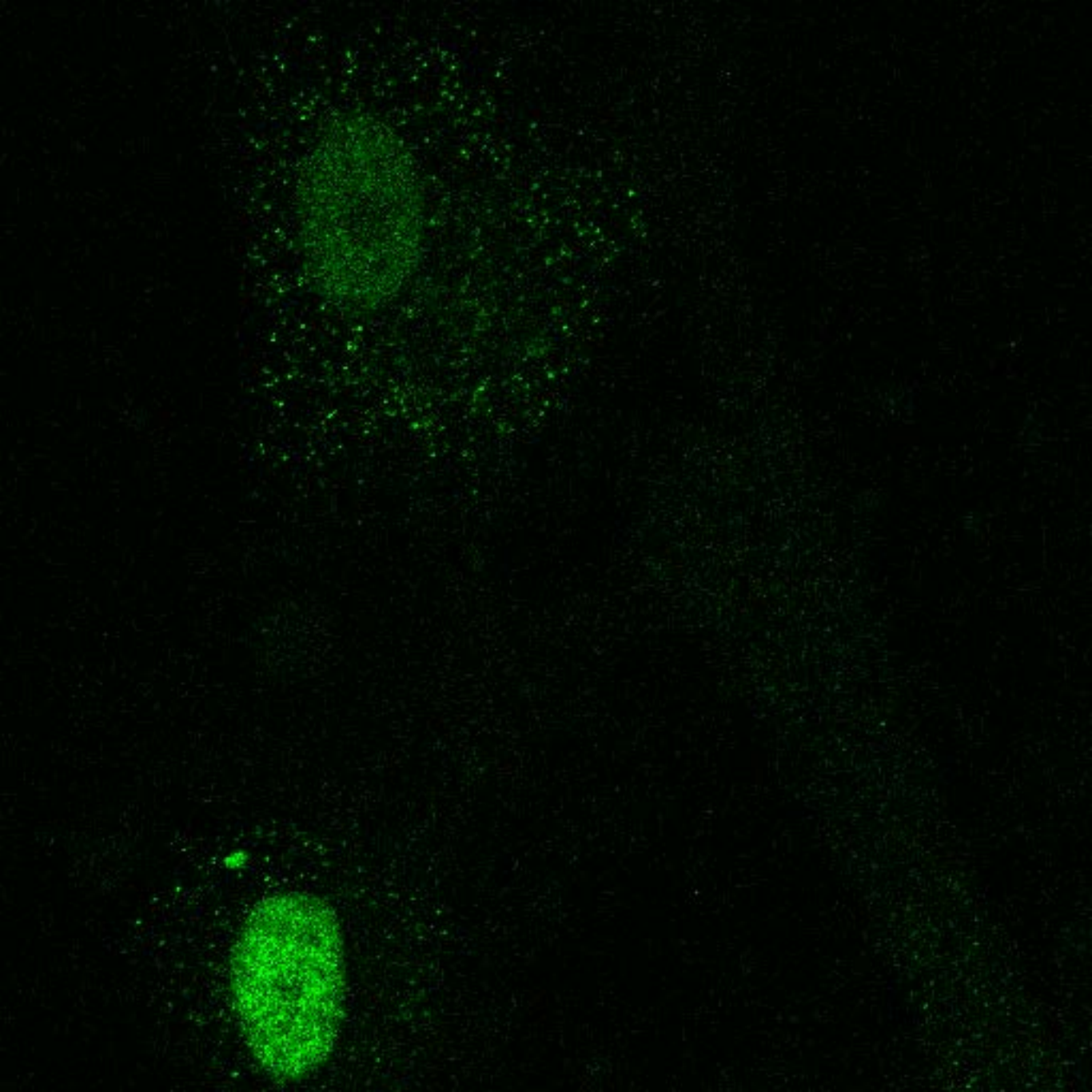

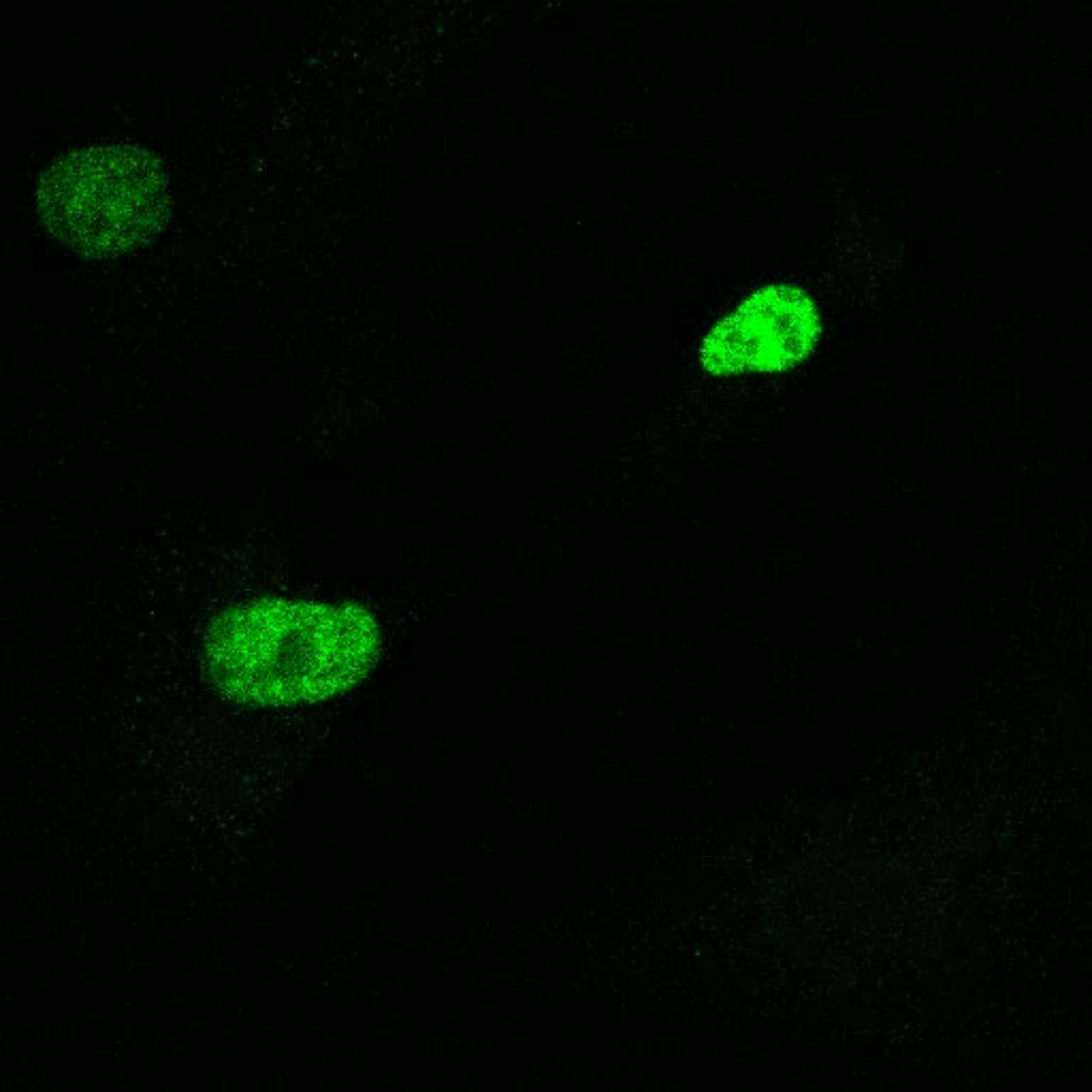

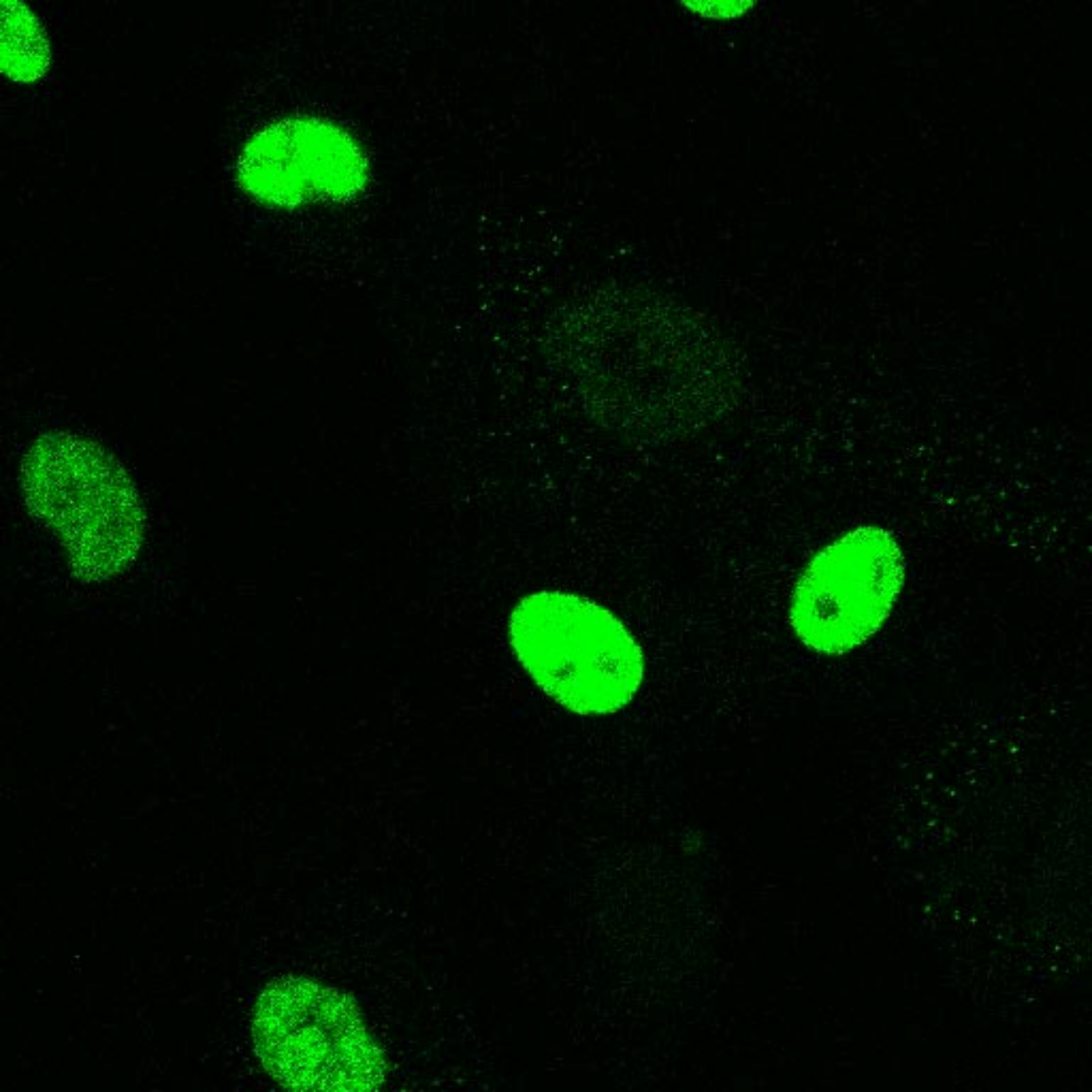

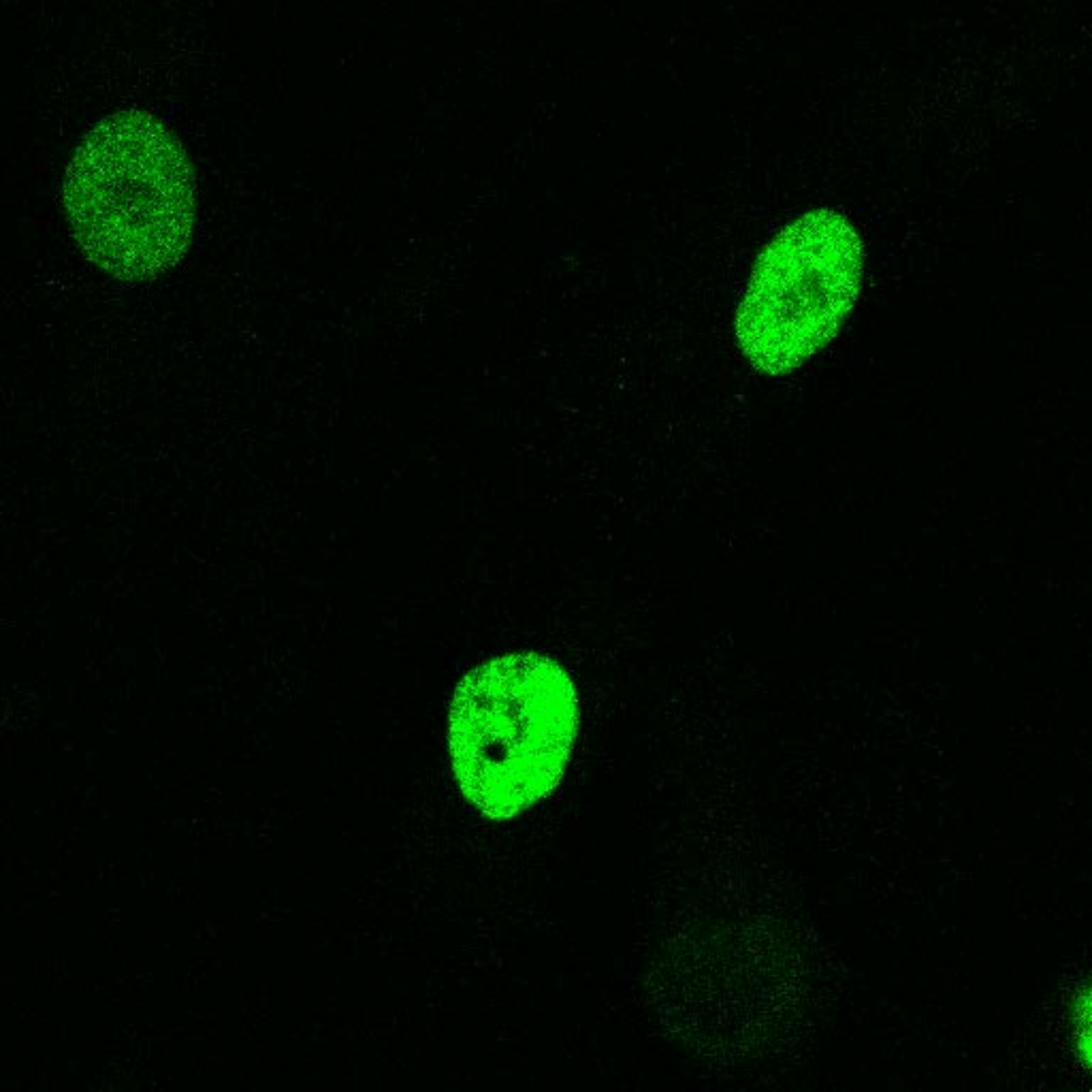

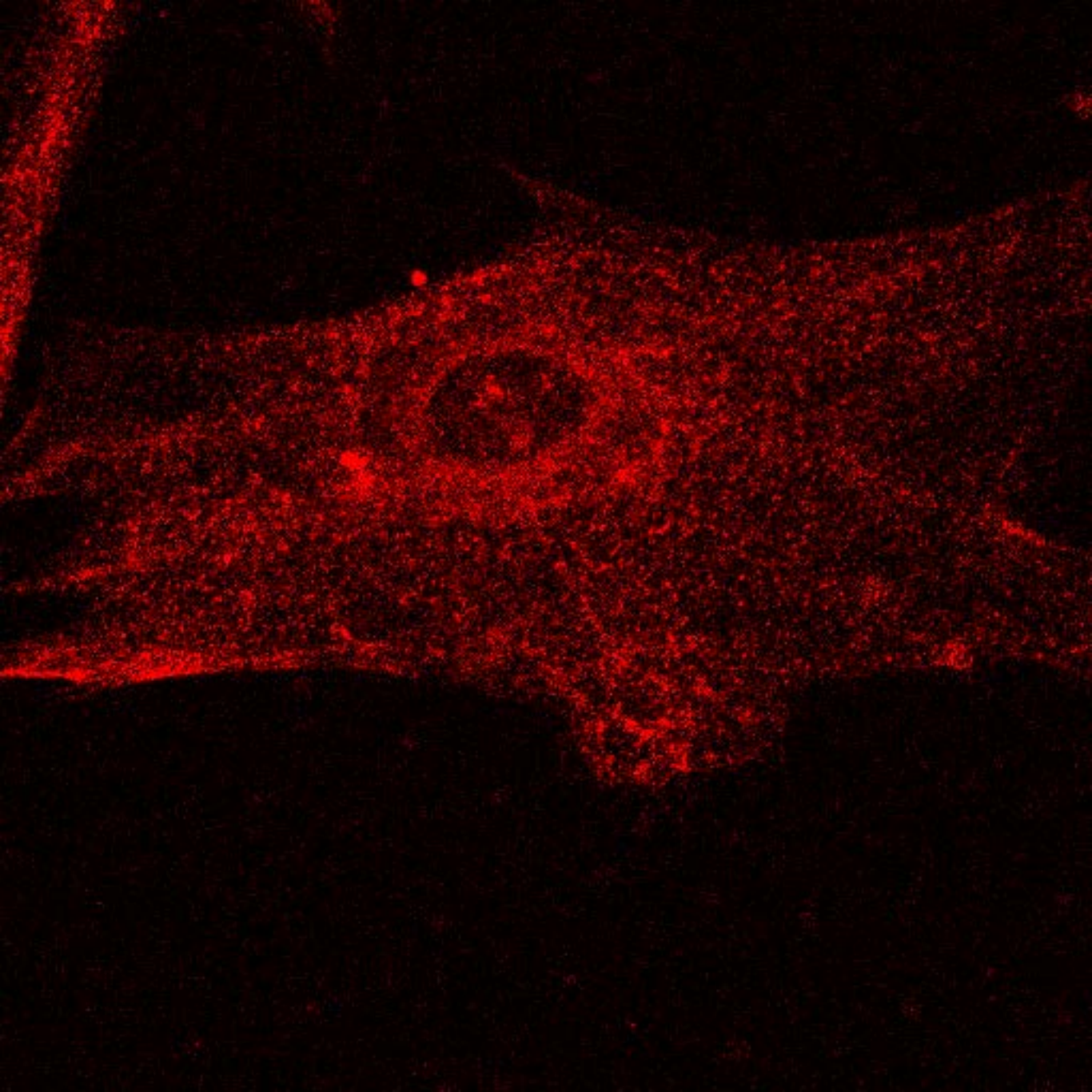













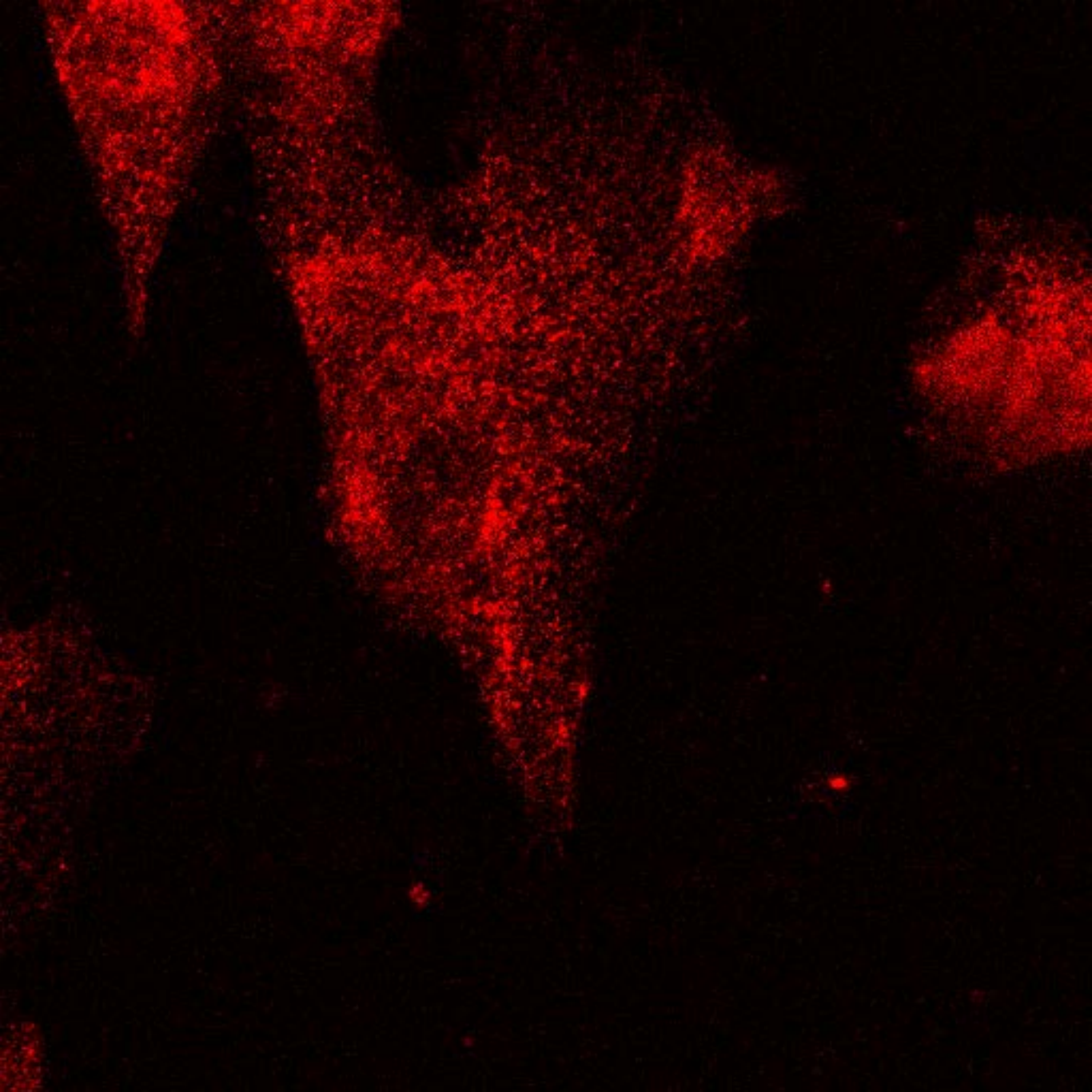

Supplement: S2 Raw images — (PDF) [file pone.0252895.s002.pdf]
